# The Role of *Escherichia coli* Shiga Toxins in STEC Colonization of Cattle

**DOI:** 10.3390/toxins12090607

**Published:** 2020-09-21

**Authors:** Christian Menge

**Affiliations:** Friedrich-Loeffler-Institut/Federal Research Institute for Animal Health, Institute of Molecular Pathogenesis, D-07743 Jena, Germany; christian.menge@fli.de; Tel.: +49-3641-804-2430

**Keywords:** Shiga toxin, verotoxin, STEC, EHEC, O157, receptor, cytotoxicity, immune response, bovine, colonization

## Abstract

Many cattle are persistently colonized with Shiga toxin-producing *Escherichia coli* (STEC) and represent a major source of human infections with human-pathogenic STEC strains (*syn.* enterohemorrhagic *E. coli* (EHEC)). Intervention strategies most effectively protecting humans best aim at the limitation of bovine STEC shedding. Mechanisms enabling STEC to persist in cattle are only partialy understood. Cattle were long believed to resist the detrimental effects of Shiga toxins (Stxs), potent cytotoxins acting as principal virulence factors in the pathogenesis of human EHEC-associated diseases. However, work by different groups, summarized in this review, has provided substantial evidence that different types of target cells for Stxs exist in cattle. Peripheral and intestinal lymphocytes express the Stx receptor globotriaosylceramide (Gb_3_ *syn.* CD77) in vitro and in vivo in an activation-dependent fashion with Stx-binding isoforms expressed predominantly at early stages of the activation process. Subpopulations of colonic epithelial cells and macrophage-like cells, residing in the bovine mucosa in proximity to STEC colonies, are also targeted by Stxs. STEC-inoculated calves are depressed in mounting appropriate cellular immune responses which can be overcome by vaccination of the animals against Stxs early in life before encountering STEC. Considering Stx target cells and the resulting effects of Stxs in cattle, which significantly differ from effects implicated in human disease, may open promising opportunities to improve existing yet insufficient measures to limit STEC carriage and shedding by the principal reservoir host.

## 1. Introduction

Bacterial colonization of the mammalian intestinal mucosa for prolonged time periods has emerged in co-evolution with the mucosal immune system. Respective bacteria (l) strains have acquired and developed mechanisms to modulate the immune response of their hosts. Heat-labile enterotoxins (LT) of *Escherichia coli* and the Cholera toxin (CT) of *Vibrio cholerae* from the group of toxins possessing a 5B-plus-A basic structure are among the best characterized bacterial immunomodulators. The holotoxins may act as potent adjuvants but their subunits may exert opposite immunological effects. Receptor-binding B subunits induce the secretion of pro-inflammatory cytokines [1] whereas receptor-mediated endocytosis of the enzymatically active A subunit results in impaired antigen processing [2] and induction of anti-inflammatory cytokines [1]. Despite assumed involvement of Toll-like receptors [3] and blood group antigens [4] in cellular binding, these immunomodulators act via certain glycolipid receptors (gangliosides, e.g., GM1) in the eucaryotic cell membrane [2].

The basic molecular structure of Shiga toxins (Stxs), secreted by Stx-producing *E. coli* (STEC; *syn.* enterohemorrhagic *E. coli*, EHEC, when proven to be human-pathogenic) resembles that of LT and CT [5]. Stxs consist of one active (StxA; 32 kDa) and five receptor-binding (StxB; 7.7 kDa) subunits [6] and can be assigned to either of two groups. The first comprises the *Shigella dysenteriae* toxin and the prototypic Stx1 of *E. coli* (also referred to as Stx1a; see Scheutz et al. [7]). In this review, the original designations in the cited publications, i.e., Stx1 and Stx2, are used. The second group comprises the prototypic Stx2 (also referred to as Stx2a) and its variants (as reviewed in [8]). Antisera against variants are partially cross-protective within but not between toxin groups [9]. Stx-encoding genes are part of the genome of lambdoid bacteriophages within the STEC genome [10]. STEC strains are often capable of producing more than one toxin type because possession of several *stx*-converting phages is common. Phages foster horizontal transmission of *stx* genes between different *E. coli* strains [11] thereby being a major driver of pathogenic *E. coli* evolution. Due to the high enzymatic activity of the A subunit catalizing the inhibition of the cellular protein biosynthesis, Stxs are primarily regarded as cytolethal toxins [12]. However, independent of their enzymatic activity, Stxs may induce a plethora of different cellular responses (as reviewed in [8]) and may even exert adjuvant effetcs by stimulating dendritic cells [13]. Stxs also exploit glycolipids (glycolipids of the globo-series) as cellular receptors [14].

Cattle and other ruminants are primary reservoirs for STEC serotypes that are frequently associated with human disease, e.g., EHEC O157:H7 [15]. Humans acquire the infection by oral route by consumption of food items or water contaminated with bovine fecal matter [15]. Since a targeted treatment for EHEC-induced human diseases like hemorrhagic colitis (HC) and hemolytic-uremic syndrome (HUS) has not yet reached clinical application [16], prevention of human infection is at highest priority. Prophylactic measures to prevent exposure is challenging as up to 82% of cattle globally shed STEC with their feces [15,17]. Cattle get infected at calf’s age by minute infection doses [18]. After initial replication in the ileum, cecum, and colon, persistent infection is established and followed by prolonged shedding of the bacteria for several months [17,19]. Strains considered to be particularly virulent to humans, like those of serotype O157:H7, preferably but not exclusively [20] colonize epithelia covering lymphoid follicles [21] and the squamous epithelium [22] at the recto-anal junction and can occasionally be found in the gallbladder [23]. STEC of other serovars evenly colonize the large bowel mucosa in numerous microcolonies [20,24,25]. In periods of low exposure (on pasture), shedding rates may temporarily drop below the detection limit for the bacteria [26], but the same STEC clone can be maintained in a single herd for months and years [17,27]. Even though STEC are shed for longer periods by calves than by adult cattle [24] and the latter harbour Stx-specific antibodies [28], a previous STEC infection does not protect from re-infection with the very same strain [24,29].

Despite the fact that colonized ruminants were identified as STEC reservoir early after discovery of this *E. coli* pathovar [15], the molecular mechanisms conferring to STEC the ability to persistently colonize this host for extended periods remain elusive. It was suggested that STEC persist in the lumen as the source of fecal shedding but the bacteria restricted to the lumen would likely be flushed from the system by digesta passage in a few days [30]. The fact that STEC can persist in the colonic digesta of some animals for many months suggests that the bacteria associate with the mucosa in some way, although in apparently extremely low numbers. In experimental infections of calves, the adhesion protein Intimin, encoded by the locus of enterocyte effacement (LEE) and pivotal for the induction of attaching and effacing lesions (AE lesions), is required for mucosal colonization [31]. Intimin as well as other components of the LEE-encoded Typ III secretion apparatus (T3SS) promotes persistence of O157:H7 and O26:H^−^ isolates in cattle and sheep [32,33,34], presumably by establishing an intimate adhesion of the bactera to the epithelial cells. Principally, experimental and natural STEC infections of cattle are asymptomatic [35,36]. Epidemiological studies [37] and experimental infections [38,39] imply that STEC are able to induce bloody diarrhea in calves under some circumstances but pathogenicity is attributed to bacterial factors of the respective strains different from Stxs [40,41]. In HC and HUS in humans and edema disease in swine, Stx-induced endothelial cell damage is the key event in pathogenesis [42,43,44]. The reported absence of Stx receptors on the bovine endothelium, at least in small vessels of the intestine, has led to the assumption that cattle are resistant to Stxs [45]. However, work by different groups, summarized in this review, has provided substantial evidence that different types of target cells for Stxs exist in cattle. Different from the immunomodulatory activities of other bacterial toxins acting as glycolipid-receptor agonists, and in contrast to the cytolethal and pro-inflammatory activity of Stxs in humans, Stxs principally act as immunsuppressive virulence factors in cattle, explaining both the lack of clinical symptoms during bovine STEC infections and the persistent character of the infection.

## 2. Target Cells for Shiga Toxins in Cattle

### 2.1. Peripheral Lymphocytes

#### 2.1.1. Receptors

The cell membrane glycolipid globotriaosylceramide (Gb_3_), also referred to as cluster of differentiation antigen 77 (CD77) when present on immune cells, acts as receptor for the B subunit of Stxs, medating endocytosis and granting the StxA subunit access to the target cells‘ cytosol (as reviewed in [8]). Stx1 and StxB1 may also trigger intracellular signalling pathways upon binding to Gb_3_/CD77 without becoming internalized by the affected cell [46].

Studies cited in this article have detected Gb_3_/CD77 by immunodecoration with a CD77-specific antibody as well as by binding studies deploying the B subunit of Stx. Of note, Gb_3_ represents a group of glycosphingolipids, sharing a common carbohydrate moiety, rather than a single, biochemically defined structure (see below). Each receptor ligand used in the studies, i.e., anti-CD77 antibody or B subunit, preferably bind to certain Gb_3_ isoforms [47]. For this reason different terms are used in the text: (a) ”CD77(-antigen)“ when the structure recognized by an antibody is referred to, (b) ”Stx receptor“ as Stx(B)-binding structure and (c) Gb_3_ when all isoforms are considered. The designation ”Gb_3_/CD77“ is used when this distinction was not applicable.

##### CD77—An Activation-Dependent Surface Antigen

Neutral glycosphingolipids, including Gb_3_, are lipoidic constituents of the cellular membrane of all mammals and the carbohydrate moieties of many glycolipids are presented on the cell surface [48]. Composition of glycolipids shows a characteristic pattern in functionally different cell types, e.g., Gb_3_ and globotetraosylceramide (Gb_4_) represent stage-specific markers for a subpopulation of terminally differentiated germinal centre B cells in humans [49]. Binding of Stxs and StxB to Burkitts lymphoma cells starts the apoptosis-inducing signalling cascade originating from Gb_3_/CD77 which synergizes with the apoptosis induction resulting from the cytosolic effects of Stx holotoxins (as reviewed in [8]).

Cells of the Bovine Leukemia Virus-infected bovine B cell line BL-3 bind anti-human CD77 antibodies [50]. However, only a minority of cells in non-stimulated cultures surface-express CD77 whereas CD77 is only detectable in intracellular compartments in the remaining cells. Surface expression is mainly restricted to cells with altered morphology indicative of affected viability (referred to as “subvital cells” in the following text). This corresponds with the observation that non-stimulated BL-3 cells resist the apoptotic activity of Stx1 [51]. Stimulation with B cell mitogens not only results in sensitation of the cells to Stx1 [51], but also in an enhanced CD77 surface expression by cells with intact morphology [50].

**Figure 1:**
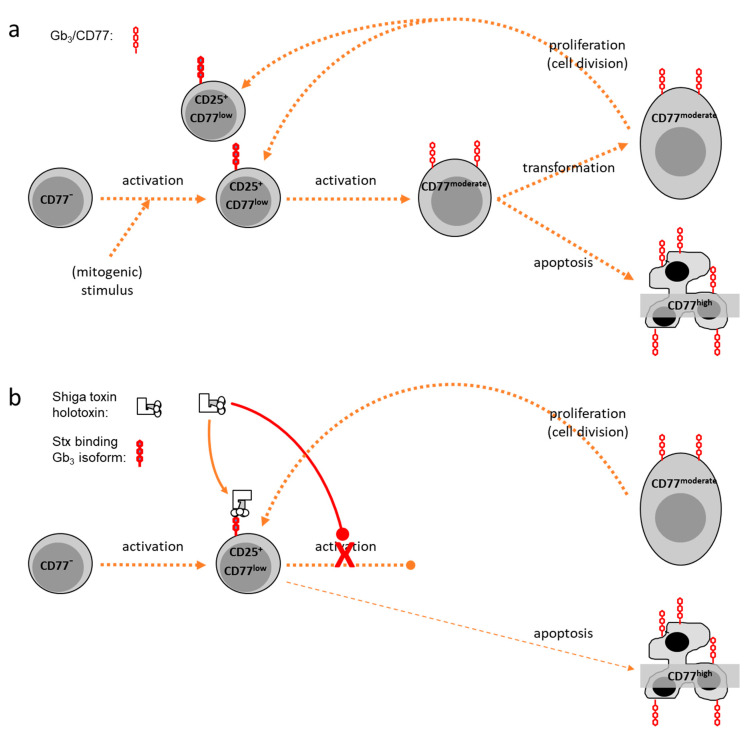
Graphical representations of activation-dependent expression of Gb_3_/CD77 (**a**) and the effect of Shiga toxins (Stxs) on transformation and proliferation of activated bovine peripheral lymphocytes (**b**). CD77 expression correlates with the activation state of bovine lymphocytes. While quiescent cells are CD77-negative or CD77^low^, activation results in formation of lymphoblasts moderately expressing CD77. CD77^high^ cells show signs of altered viability. Gb_3_ isoforms acting as high affinity Stx receptors are only transiently expressed in early stages of the activation process in the transition phase from the CD77^low^ to the CD77^moderate^ state. Lymphocytes affected by Stxs either remain in this state or initiate the apoptotic process accompanied by a transient increase in CD77 expression. Cells that went through the entire activation process from to CD77^high^ stages become refractory to Stxs but may regain Stx-sensitivity after division and reentering the activation cycle.

Similarly, bovine peripheral blood mononuclear cells (PBMC) fail to bind anti-CD77 unless stimulated in culture [50]. Particularly promoted by polyclonal (mitogenic) stimulation, CD77 becomes increasingly expressed on the lymphocytes‘ surface [52] as a function of time and strength of the stimulus. Yet, kinetics of CD77 expression vary with the morphology of the cells, which reflects activation status and viability. Enlarged blast cells, developing from non-blast cells after stimulation, comprise the largest portion of CD77^+^ cells [50]. The number of CD77^+^ blasts peaks at day 3 to 4 of culture while the portion of subvital CD77^+^ cells continues to rise. The latter population, exhibiting the morphology of apoptotic cells (reduced cell size, increased granularity) [53], expresses highest numbers of CD77 molecules on their surface, leading to a cumulation of CD77^high^ cells in bovine PBMC cultures in the absence of Stx. Nevertheless, CD77 expression does not correlate with signs of cellular death but seems to indicate an intermediate activation state of bovine lymphocytes in vitro. While quiescent cells (“non-blasts”) are CD77-negative or CD77^low^, (mitogenic) activation induces elevated levels of CD77 such that up to 60% of blasts moderately express CD77. Disappearance of these CD77^moderate^ cells in the cultures is accompanied by a rise in subvital CD77^high^ cells. Similar to BL-3 cells, levels of CD77 surface expression up to the CD77^moderate^ state seems to parallel the activation process of the cells. At the CD77^moderate^ state of activation, cells either survive and divide, with the daughter cells thereby becoming CD77^low^ non-blasts again or undergo apotosis followed by a further increase of surface CD77 expression (CD77^high^) (Figure 1a).

This pattern of CD77 expression resembles human B cells. CD77^+^ human B cells exhibit the phenotype of activated cells but are negative for classical activation markers CD23, CD25, and CD71 and show characteristics of apoptotic cells [53]. However, cells are not destined to undergo cellular death but may be rescued by Interleukin (IL-) 4 and CD40-ligand treatment [54]. Surviving B cells down-regulate CD77 expression and become CD23^+^ again [54]. Although bovine CD77^+^ lymphocytes partially coexpress CD25 [55], CD77 represents an activation marker indicative of certain stages in the activation process, when expressed by bovine lymphocytes upon stimulation in vitro [50] or in vivo [50,56].

CD77 is expressd by several bovine lymphocyte subpopulations (γδT cells, CD4^+^ T cells, CD8α^+^ T cells, B cells) [50], but subsets differ in their expression patterns. Almost all CD8α^+^ T cells, being particularly sensitive to Stx1 [51], co-express CD77 in high numbers, whereas the number of positive cells as well as the level of CD77 surface expression on less sensitive CD4^+^ T cells and B cells is comparably low. In humans, CD77 expression on lymhocytes was initially associated with oncogenic transformation of Burkitt-Lymphoma cells [57]. In healthy individuals, CD77 indicates a subpopulation of germinal centre cells [53]. CD77 is a weak marker for proliferating centroblasts engaged in the somatic hypermutation process but no longer expressed by centrocytes involved in the class switch recombination process [58]. Although several human lymphoid pre-B and T cell lines and myeloid cell lines were initially considered to lack CD77 [46,59], Ramegowda and Tesh [60] confirmed results by Kniep et al. [61], who had chemically detected Gb_3_ molecules in human myeloid cells at later differentiation stages. CD77 expression by T cells, on the contrary, appears to be unique to cattle and is of considerable importance for understanding the evolution of STEC as a bovine adapted subset of *E. coli*. This particular holds as expression of CD77 by bovine lymphocytes is not restricted to in vitro conditions: CD77 is present on the surface of many B cells in mesenteric lymphnodes as well as on the surface of CD4^+^ and CD8α^+^ T cells from these tissues [50] and from mucosal sites [56].

##### Biochemical Characterisation of Bovine Gb_3_/CD77

The increase of mitogen-induced CD77 expression on the surface of cultivated bovine PBMC is paralleled by an overall increase in the neutral glycolipid content of the cells, in particular by an increase in ceramide trihexosides [50]. While neutral glycolipids in unstimulated PBMC mainly consist of ceramide monohexosides and only traces of di- and trihexosides, stimulated PBMC contain ceramide mono- and -trihexosides in equivalent amounts with the latter having a Gal(α1-4)Gal(1-4)Glc(1-1)Cer structure, i.e., globotriaosylceramide, Gb_3_. The lipid portion of ceramide mono- and trihexosides consists of C18 sphingosine and fatty acids of various length. Although C16:0 is the dominating fatty acid species in all ceramide mono- and trihexosides of bovine lymphocytes, C22:0 and C24:0 fatty acids are also incorporated. Of note, a considerably higher portion of ceramide trihexosides of stimulated PBMC contain fatty acids with more than 20 carbon atoms. The functional consequence of this finding is discussed below.

Seemingly, the activation-dependent expression of Gb_3_/CD77 on the surface of bovine lymphocytes is steered by several factors. The biochemical detection of Gb_3_ in unstimulated lymphocytes at a stage cells lack binding sites for anti-CD77 on their surface as well as the fact that CD77 is mainly detectable intracellularly in BL-3 cells [50] can be explained by a specific feature of glycosphingolipids, termed crypticity [62]. It may result from the exclusive presence of Gb_3_/CD77 in intracellular compartments as well as from masking of the relatively small extracellular carbohydrate portion of the molecules by intrinsic membrane proteins. In addition to a possible cellular redistribution of Gb_3_/CD77, stimulation of bovine lymphocytes clearly increases the total amount of ceramide monohexosides and Gb_3_ accompanied by shifts in the fatty acid composition of their lipid portion, implying that induction of Gb_3_/CD77 surface expression mainly results from de novo synthesis of glycosphingolipids. A transient activation of the respective glycosyltransferases is also implicated in shifts of the glycolipid composition during differentiation of human B Cells [49].

##### Binding and Internalization of Shiga Toxin and Its B-Subunit

In line with the finding that resting bovine lymphocytes fail to bind anti-CD77, the cells also lack detectable numbers of binding sites for Stx1 holotoxin or StxB1 subunit unless stimulated by mitogens for several days [63]. Binding occurs to several lymphocyte subpopulations with a pattern reflecting the subsets susceptibility to Stx1 (B cells > CD8α^+^ T cells > CD4^+^ T cells > γδT cells). Interstingly, binding capability for StxB1 does not directly correlate with the cells’ ability to bind anti-CD77. Early during the activation process of bovine lymphocytes in vitro, a population of CD77-negative cells can bind StxB1, whereas at later time points a population of CD77^+^ cells unable to bind StxB1 predominates. Pre-incubation with StxB1 may hinder anti-CD77 from binding and high concentrations of StxB1 are able to remove anti-CD77 from its binding sites [63]. During the process of bovine lymphocyte activation, three states exist: (a) cells with Gb_3_ molecules with particular affinity for StxB1, (b) cells with Gb_3_ molecules with particular affinity for anti-CD77 and (c) intermediate states with Gb_3_ molecules capable of binding both ligands with equal affinity. StxB1 and anti-CD77 monoclonal antibody 38.13 both specifically recognize the terminal galabiose residue (Gal(α1-4)Gal) of Gb_3_ molecules [57,64], but the recognized epitopes slightly differ from each other [47]. Binding of ligands is also impacted on by the membrane environment of Gb_3_ and the composition of its lipid moiety [47]. Only in early stages of the activation process, bovine lymphocytes predominantly harbour Gb_3_ molecules containing C16 fatty acids [50], known to exhibit a significantly higher affinity for Stx1 as compared to other ceramide trihexosides [65]. This likely explains why the cells are particularly prone to bind StxB1 and are particularly sensitive to Stx1 only at this stage.

Ribosomes represent the prime target structure for the enzymatic activity of Stx, which only can be reached by the toxins if internalized, e.g., via clathrin-coated pits after clustering of Stx binding sites (as reviewed in [8]). Although the portion of bovine lymphocytes expressing high affinity Gb_3_ molecules for Stx1 is highest at the initiation of activation, at least a portion of these molecules is able to mediate internalization of Stx1 within 30 min even if cells are pre-cultured and activated for up to 4 days [63]. This points to the existence of a Gb_3_/CD77 coupled translocation mechanism in bovine lymphocytes which is active even in advanced activation stages.

#### 2.1.2. Effects of Shiga Toxins In Vitro

In vitro and in vivo studies unveiled immunomodulatory effects of Stxs in different species [59,66,67] but the molecular basis for this is still poorly understood. The presence of Stx1 in the picogram concentration range in bovine PBMC cultures dramatically reduces the number of CD77^+^ cells with blast and non-blast morphology [55]. In the early phase of cultivation, however, the number of CD77^+^ cells with the morphology of quiescent cells is increased by Stx1 and CD77^+^ lymphocytes remain detectable for several days. It is tempting to assume that Stxs primarily kill Gb_3_/CD77^high^ cells as it was described for several cell lines [68], but metabolic depression in Stx-treated bovine PBMC cultures is neither accompanied by significant increases in apoptotic nor in necrotic cells [51,52]. Stx1 also reduces the portion of quiescent non-blast cells exhibiting low to moderate CD77 expression levels later during cultivation [50]. Considering that bovine lymphocytes express high affinity Stx receptors only transiently in early stages of the activation process [63], Stx1 only targets a comparably low number of cells in the transition phase from the CD77^low^ to the CD77^moderate^ state. Affected cells then either remain in this state or initiate the apoptotic process accompanied by a transient increase in CD77 expression as implied by the observation of a transiently increased number of CD77^+^ non-blasts in Stx1-supplemented bovine PBMC cultures [55]. The magnitude of Stx1 effects was similar when toxin was added not earlier than day 3 of culture, i.e., in a phase with many lymphocytes exhibiting a CD77^high^ phenotype. This implies that bovine lymphocytes become refractory again once they went through the entire activation process from CD77^low^ to CD77^moderate^ to CD77^high^ stages (Figure 1b).

Different lymphocyte subsets vary in their sensitivity but Stx1 reduces the portion of CD77^+^ cells in all subsets of bovine PBMC in vitro. By contrast, Stxs seemingly only target the B cell compartment in humans [53,59]. Because in cattle, the toxin’s effect focusses on lymphocytes in intermediate stages of the activation process and less on lymphocyte subsets, STEC by secreting Stxs primarily block the development of an active adaptive immune response rather than down-regulating an established one.

The pathogenesis of HUS in humans is mainly driven by the local induction and release of pro-inflammatory cytokines, like Tumor necrosis factor alpha (TNF-α), by Stxs (as reviewed in [8]). Supplementation of cell culture medium with bovine TNF-α neither affects CD77 expression by bovine lymphocytes nor does it mimic the effect of Stx1 [55]. Interferon alpha (IFN-α) binds to its receptor (IFNAR) only if it is present in its high affinity form, complexed with Gb_3_/CD77 [69]. Despite the partial homology between the bovine IFNAR and StxB1 [69], addition of IFN-α further increases the inhibitory effect of Stx1 on bovine lymphocytes, implying that both molecules utilize different signal transduction pathways [55]. Although Stx1 induces and prolongs expression of bovine IL-2 receptor (CD25) [51], exogeneous IL-2 does not prevent the inhibitory effect of Stx1 [55], arguing against a Stx-induced blockage of paracrine IL-2 secretion as described for human lymphocytes in response to the *lifA* gene product (see below) of enteropathogenic *E. coli* [70,71]. Effects of Stxs on bovine peripheral lymphocytes seem to primarily result from direct impact on the cells.

Binding of StxB subunit, lacking the holotoxin’s enzymatic activity, to cellular membranes may induce signal transduction pathways (as reviewed in [8]). Incubation of bovine PBMC with recombinant Shiga toxoids devoid of the enzymatic activity of wild type Stx1 and Stx2 (see below) neither influences the percentage of lymphocytes expressing CD77 nor the overall subset composition [72]. Even though the molecular mechanism by which Stxs induce cell death in a variety of cell lines and primary cells is well understood (as reviewed in [8]), the molecular basis of the immunomodulatory effects of wild type Stxs to bovine immune cells is not entirely clear. In most cells, Stxs primarily inhibit protein synthesis by acting on the 23S rRNA incorporated in ribosomes [73]. THP-1 cells show an up-regulation of TNF-α upon treatment with Stx1 [74], an effect traced back to the ribotoxic stress response triggered by the enzymatic activity of Stxs. It would also be plausible that cross-linking of CD77 molecules on the cellular surfaces by the multivalent 5B-plus-1A structured Stxs initiates cellular responses independent of the enzymatic activity as toxin binding is sufficient to induce apoptosis in sensitive cell lines [75]. Studies with Shiga toxoids, though, suggest that the enzymatic activity is required for the immunomodulating effect of Stxs in cattle [72]. Evidence exists that bovine lymphocytes also respond to StxB [63] but markedly high concentrations are required. In other in vitro systems, Stxs sensitize cells to membrane-derived signals by inhibition of protein synthesis. Such signals may then be triggered by very low amounts of StxB and initiate the apoptosis programm [46,76]. Stx1 fails to induce apoptosis in bovine lymphocytes but still may damage 28S rRNA and trigger a ribotoxic stress response. Signals derived from binding of StxB (or the pentamer) to surface-expressed Gb_3_/CD77 may then act in concert with the induction of early response genes after internalization of the Stx holotoxin, synergistically exerting suppressive effects on bovine lymphocytes.

### 2.2. Intestinal Intraepithelial and Mucosa-Associated Lymphocytes

Although STEC were occassionally isolated from intestinal lymphoid tissues [24], and in vitro studies suggest that STEC O157:H7 may invade non-intestinal bovine epithelial cells [77], STEC are generally considered not to be invasive but to only colonize mucosal surfaces along the bovine intestinal tract. Stxs produced and secreted by mucosal STEC microcolonies affect the immune response of bovine hosts at the systemic level [29,78], strongly implying that Stxs get translocated across the intestinal wall also in the reservoir host with intraepithelial lymphocytes (IEL) as the first adaptive immune cells to experience the toxins.

#### 2.2.1. Stx Receptor Expression by Subsets

CD77 is present on the surface of all major IEL subsets including αβT cells and γδT cells isolated from the ileal mucosa of cattle but with varying expression levels [56]. The majority of CD77^high^ ileal IEL (iIEL) are activated, maturated T cells coexpressing CD3, CD6, ACT-2 and CD8α. CD8β^+^ T cells and γδT cell subsets (WC1^+^, TcR1-N7^+^) also show strong surface expression of CD77 whereas CD4^+^ T cells and B cells (CD21^+^, membrane IgM^+^) express CD77 only at low levels. Like peripheral lymphocytes, some bovine IEL are capable of binding anti-CD77, some bind rStxB1 and some both ligands. The predominance of anti-CD77 binding isoforms of Gb_3_ points to an activated cellular state, supported by the fact that many CD77^+^ bovine iIEL coexpress the activation marker ACT-2 [79]. CD77 is particularly expressed by TcR1-N7^+^ and WC1^+^ subsets of γδT cells coexpressing the CD8αα homodimer. Main functions ascribed to CD8αβ^+^ αβT IEL in mice and men is the lysis of infected cells whereas γδT IEL (and probably CD8αα^+^ αβT IEL) govern the regeneration of the epithelial cell layer. Of note, a Stx-induced damage of epithelial cells resulting in both alteration of local immune responses as well as of the renewal of the intestinal epithelium has been associated with the persistent character of bovine STEC infections [80].

Cells present in lymphoid aggregates within the lamina propria, particularly of the ileum, possess Stx binding sites [81], but the meaning of this remains to be unveiled.

#### 2.2.2. Functional Implications of Stx Receptor Expression In Vitro

Bovine iIEL cultures respond to the presence of Stx1 by a reduced transformation rate of non-blast cells to blast cells, particularly affecting CD77^+^ cells similar to PBMC. Interestingly, in vitro stimulated bovine iIEL exhibit a CD77 expression pattern different from the cellular distribution ex vivo. Upon cultivation, equal portions of CD8α^+^ and CD8β^+^ iIEL express CD77 while the highest portion of CD77^+^ cells becomes detectable within the CD4^+^ T cells and CD21^+^ B cells, implying that all subpopulations of bovine iIEL are readily capable of expressing CD77.

Bovine iIEL possess a natural killer cell activity and are the source of chemokines (IL-8, IP-10, MCP-1) but Stx1 affects neither of these functions [56,82]. These in vitro findings support data from in vivo studies showing that Stxs are not implicated in the induction of intestinal inflammatory responses in the course of STEC infections in calves [35].

In vitro, Stx1 also does not affect the transcription of a number of prototypic T helper cell (T_H_) cytokines (IL-2, Interferon gamma (IFN-γ), IL-10, TGF-β). A remarkable exception is a strong increase of the cellular content of mRNA for the T_H_2 cytokine IL-4, which becomes detectable 4 h after addition of minute amounts of Stx1 to bovine iIEL cultures and reaches its maximum after 6 to 8 h [56,82]. This effect may be the result of an increased *IL-4* transcription [83]. However, Stx, by activating the p38 MAP kinase signalling pathway [84,85], may also prolong the half-life of mRNA species encoding for selected cyto- and chemokines [74,86]. Indeed, the translation-dependent mRNA degradation in mammalian cells occurs transcript-specific [87]. Nevertheless, the increase in *IL-4* mRNA is not accompanied by a detectable increase in IL-4 protein in bovine iIEL [82]. This may be either because the amounts of produced protein is below the detection limt [86] or because the predominance of IFN-γ in bovine iIEL cultures leads to reciprocal down-regulation of a T_H_2-biased response [88] masking the Stx effect. The enzymytic activity of the Stx1 holotoxin is indispensable to induce the increase in *IL-4* mRNA because neither StxB1 [82] nor Shiga toxoids [72] induce this effect. Stx1 acts at very low concentrations (equivalent to 7 verocytotoxic doses 50% per ml [CD_50_/_mL_]) leaving the ability of iIEL to synthezise IFN-γ unaffected [82]. Bitzan et al. [89] observed that Stxs may increase the amount of certain transcripts in bovine endothelial cells at concentrations below those required for a detectable inhibition of cellular protein biosynthesis implying that the discrepancy between data on *IL-4* mRNA and on IL-4 protein may not be simply due to Stx-induced inhibtion of protein synthesis.

Bovine CD4^+^ T iIEL can be activated to express Gb_3_/CD77 and thereby rendered susceptible to Stx1. IL-4 is typically produced by the T_H_2-determined subpopulation of CD4^+^ T cells. However, the Stx-induced increase in *IL-4* mRNA in bovine iIEL could not be attributed to a certain cell population thus far. Ribosome-inactivating toxins like misteltoe lectins equally induce the intracellular expression of IL-4 in CD4^+^ as well as in CD8^+^ T cells [90]. Induction of *IL-4* transcription is then associated with induction of apoptotic pathways [90]. The Stx-induced increase in *IL-4* transcripts in bovine iIEL cultures occurs independent of the induction of apoptosis [82]. As a consequence, bovine IEL responding to Stxs in the course of STEC colonization of the intestinal mucosa remain to act as part of the mucosal immunological network. Interestingly, IL-4 specifically inhibits the stimulation of human CD8^+^ IEL without affecting the proliferation of peripheral CD8^+^ T cells [91]. Even though an increase in IL-4 protein could not be detected in Stx-exposed bovine iIEL thus far, the depletion of CD8^+^ IEL during experimental STEC infections of calves [92] may, at least in part, be explained by a subtle but biologically relevant production of IL-4 triggered by Stx.

### 2.3. Macrophages

Stx-induced pro-inflammatory responses of macrophages (Mø) are central to the pathogenesis of human disease [44]. Despite the fact that only one third of bovine blood monocyte-derived Mø express CD77 on their surface, CD77 is present in intracellular compartments of the vast majority of the cells [93]. A significant portion of cells is capable of binding the StxB1 at their surface. Cameron et al. showed that Stx2 stimulated a transient increase in mitogen-activated protein kinases activity in bovine Mø [94] with similar kinetics to human monocytes [95]. In these studies, Stx2 stimulated the release of TNF-*α* protein from bovine cells only at relatively high concentrations (100 ng/mL), while human monocytes still responded to 100 pg/mL. By contrast, 200 CD_50_/_mL_ of Stx1 (equivalent to 120 pg/mL of purified Stx [96]) were sufficient to significantly down-regulate the surface-expression of CD14, CD172a and co-stimulatory molecules CD80 and CD86 within 4 h of incubation in a study conducted by our group [93]. At the same concentrations, Stx1 increased the numbers of transcripts for *IL-4*, *IL-6*, *IL-10*, *IFN-γ*, *TNF-α*, *IL-8*, and *GRO-α*, but not for *IL-12*, *TGF-ß*, *MCP-1*, and *RANTES*. Such a mixed pattern consisting of T_H_1- and T_H_2-associated cytokines is reminiscent of alternatively activated M2-Mø [97] developing following activation in the presence of IL-4 and consisting of several functionally different subtypes [98]. The pattern induced by Stx1 most closely resembles the phenotype of type II-activated Mø, also referred to as regulatory Mø [98]. These cells typically express both pro- and anti-inflammatory cytokines and chemokines, as well as IL-10, but fail to express IL-12.

Such a pattern is also found in presumptive mucosal Mø isolated from the bovine colon and cultured in the presence of Stx1 [99]. CD14^+^ mesenchymal cells in primary bovine colonic crypt cell cultures surface-express high levels of CD77 and are sensitive to modulation by Stx1. Morphological features, markers and cytokine expression profiles and the fact that Stx1, in conjunction with lipopolysaccharide (LPS), induces a shift towards increased *IL-10* transcription [99], suggest that the predecessors of these cells belong to type II intestinal macrophages. Because Stx1 rapidly and specifically induces transcription of *IL-4* in bovine iIEL [56,82], the combined effect of Stx1 on Mø and IEL beneath the site of epithelial colonization in the bovine intestine may skew the local immune system towards T_H_2, thereby imprinting on the subsequent inflammatory and adaptive immune response.

Of note, only a small subset of CD77^+^ cells in the lamina propria of the bovine distal colon in close proximity to crypts coexpresses CD14 [99]. CD77^+^ cells are infrequently located in subbasilar lymphoid aggregates within the lamina propria, and occasionally, scattered individual CD77^+^ cells are present in the lamina propria not associated with lymphoid aggregates. CD14^+^ cells are frequent within the epithelium of mucosal crypts, as well as the surface epithelium. Individual CD14^+^ cells reside within the lamina propria, particularly focused in lymphoid aggregates. Cells coexpressing CD77 and CD14 are absent from the epithelium of the mucosal crypts or surface epithelium but present in low numbers in the lamina propria between the colonic crypts. Thus, several CD77^+^ mesenchymal cell types exist in the bovine colonic mucosa that are potentially sensitive to Stxs in situ and await further functional characterization.

### 2.4. Granulocytes

In humans, neutrophilic granulocytes appear to be implicated in the early, local events in the course of STEC infections, which eventually result in the pathogenesis of HC and HUS [44]. Stxs delay the onset of apoptosis in human neutrophils [100] and promote the generation of reactive oxygen metabolites, contributing to the tissue damage in the intestinal mucosa [101]. Stxs also induce chemoattractant release from epithelial cells [86,102] and the subsequent transmigration of neutrophils across the epithelial layer [103]. Resulting intestinal inflammatory responses are a presumptive prerequisite for sufficient amounts of Stxs to be resorbed from the intestinal lumen [44]. Evidence exists that polymorphonuclear leukocytes function as toxin transporters to the sensitive organs like the kidneys [104].

Granulocytes, in the first place represent effector cells of the innate immune system implicated in limiting mucosal colonization by bacteria [105]. Granulocytes infiltrate the colonic mucosa of STEC-infected calves [106], but their relevance for the course of bovine STEC infections is to be determined. The lack of overt intestinal inflammatory signs in STEC-colonized adult ruminants imply that granulocytes are not attracted to the mucosa and become activated here. Indeed, inflammation induced by certain STEC strains in an intestinal loop model with calves are independent of Stx expression [35]. The hypothesis that granulocytes in humans and cattle fulfill different roles in the pathogenesis of STEC infections is supported by the fact that bovine peripheral blood granulocytes, even after stimulation with LPS, known to increase the expression of other surface markers like CD11b [107], fail to express Stx receptors [108]. An age-dependency of receptor expression, described for the intestinal epithelial cells in rabbits [109] was ruled out by testing granulocytes of 1–3 week old calves. Granulocytes, having passed the blood-milk barrier and exerting altered cellular functions [110], also lack CD77. Accordingly, Stx1 neither affects the viability of bovine granulocytes, nor their phagocytic activity and reactivity [108]. The resistence of bovine granulocytes to Stxs may be another aspect explaining why STEC infections and carriage in adult cattle do not result in any clinical signs.

### 2.5. Endothelial Cells

Endothelial cells in the microvasculature of the gastrointestinal tract, the kidneys, and the central nervous system are the principal targets for Stxs in the pathogenesis of human STEC infections. Immunohistological studies with the respective tissues from bovines, by contrast, failed to detect Stx binding sites on endothelial cells [45,81]. This has led to the assumption that cattle are tolerant reservoir hosts for STEC [45]. However, primary bovine umbilical vein endothelial cells surface express CD77 in vitro [111]. Stimulation with LPS dramatically increases surface expression, presumably by both, de-novo synthesis of CD77 molecules and relocation of binding sites from intracellular compartments to the cellular surface. Stx1 neither exerts cytholethal effects nor significantly alters expression of *IL-8*, *GRO-α*, *IP-10*, *MCP-1*, *RANTES*, *TGF-ß* and *IL-12* in these cells, irrespective of the absence or presence of LPS [111]. In quiescent bovine aortic endothelial cells, Stxs, but not StxB, induces concentration-dependent (0.1–10 nM) increases in steady state prepro-endothelin-1 (ET-1) mRNA transcript levels, an effect maximal at 12–24 h [89]. Stxs increase preproET-1 mRNA transcript levels at concentrations that have trivial effects on nascent DNA, RNA, and protein synthesis, whereas endothelin converting enzyme-1 and endothelial constitutive NO synthase mRNA transcript levels remain unchanged. It cannot be ruled out, therefore, that subtile changes in endothelial cell functions induced by sublethal concentrations of Stxs participate in molecular host–pathogen interactions of STEC with their cattle host.

### 2.6. Intestinal Epithelial Cells

Presence of Gb_3_/CD77 in intestinal epithelial cells (EC) in the crypts close to the submucosa was found in primary cultures as well as in frozen tissue sections from the bovine small and large intestine [81,112,113] and fetal bovine intestinal EC cultures [52]. Stx1, but not Stx2, binds to colonic EC at low levels of differentiation [113] whereas differentiated EC, that underwent proliferation and migration to the luminal surface, lack Stx receptors [81]. A subset of cultured colonic EC can bind anti-CD77 as well as StxB1 at the surface, but the majority of CD77 is located intracellularly in juxtanuclear areas in these cells [99]. Bovine colonic EC contain different isoforms of Gb_3_ [81], some of which are not located in lipid rafts but randomly distributed in the membrane [112]. The combination of Gb_3_ isoform, membrane raft/non-raft distribution and intracellular route of transportation is assumed to result in lysosomal degradation of the Stxs and to prevent them from being translocated to the ER as in Stx-sensitive cells [114]. As a result, bovine intesinal EC resist the cytotoxic activity of Stxs [99,112]. Neither viable STEC O157:H7 nor Stx-containing bacterial extracts are enterotoxic, i.e., caused fluid accumulation, in ligated ileal loops in newborn calves [45].

In vitro, bovine colonic EC show an unaltered expression of chemokines GRO-α, IL-8 and RANTES in the presence of Stx1. Nevertheless, Stx1 slightly induces transcription of the gene encoding mono- and lymphocytotropic MCP-1 [99] indicating that bovine colonic EC possess some responsiveness to Stx1. Human Mø also transport Stxs to lysosomal compartments following Gb_3_/CD77-mediated endocytosis [115] and are refractory to the cytolethal effetc of Stx1 [116]. These cells still respond to Stx1 by an increased expression of pro-inflammatory cytokines [116,117].

Further evidence that bovine EC are relevant target cells for Stxs is provided by studies linking colonization and EC proliferation. It was observed earlier that, after experimental inoculation with a *stx*-negative *E. coli* O157:H7 strain, cattle with slower rates of intestinal cell proliferation in the cecum and the distal colon were *E. coli* O157:H7 culture positive significantly longer than cohort cattle with faster cell proliferation rates [30]. Recently, Fitzgerald et al. showed that both, Stx2a and Stx2c, can restrict cellular proliferation within bovine crypts (organoids) independent of cell death and apoptosis with evidence of increased activity in the presence of Stx2a, a toxin type contributing to increased shedding and animal-to-animal transmission of *E. coli* O157 in cattle [80].

The effect might come from direct targeting of EC or indirectly via affecting the release of mediators and growth promotors from adjacent cells. Of note, intestinal EC not only represent target cells for Stxs but, in the first place, are the only cells of the bovine host in direct contact with STEC bacteria. An intense molecular interplay between different STEC strains and their virulence gene repertoire and host responses has to be considered [35,118,119,120]. STEC possessing the LEE locus attach to bovine colonic EC and, within 6 h, induce an increase in mRNA transcripts for *IL-8*, *GRO-α*, *MCP-1*, *RANTES*, and *IL-10* [121]. Induction is more prominent as with enteropathogenic *E. coli* (EPEC) possessing the LEE and inducing AE lesions but lacking *stx*. A mitigated inflammatory EC response to EPEC, compared to STEC, can be traced back to persistent NF-κB inhibition by a LEE effector protein, resulting in a block of *IL-8* transcription and secretion within hours post-infection [122]. The differences in the effects by STEC and EPEC appear to be independent of Stx, therefore. Different STEC strains, however, vary in their interaction with bovine intestinal EC in quantitative and qualitative terms. The O104:H4 enteroaggregative *E. coli* (EAEC)/EHEC hybrid strain, which caused the severe German HUS outbreak in 2011, significantly induces *IL-6* transcription in bovine small and large intestinal EC while other STEC strains, capable of colonizing cattle at least transiently, induced pro-inflammatory mediators to a lower extend [123]. When primary bovine Mø were cultured with supernatants from the aforementioned cultures after co-incubation with *E. coli* strains, conditioned medium from outbreak strain co-cultures was uniquely capable of inducing a strong proinflammatory activity in bovine Mø, i.e., a dramatic up-regulation of *MCP-1* and *iNOS* transcription which indicates a strong chemotactic and bactericidal activity exerted by the cells [123]. The complex interplay of STEC strains possessing different virulence gene patterns including but not restricted to *stx* genes [124] awaits to be fully elucidated. It is highly likely, though, that Stxs interfere with the signalling network between different cells in the immediate environment of STEC microcolonies in the bovine intestine including the targetting of a subset of EC. Such a concept of Stx toxicity depending on epithelial-mesenchymal cross-talk, in which mesenchymal cell damage causes epithelial cell damage, was recently described to characterize tissue responses to Stxs in human intestinal organoids [125].

## 3. Intestinal Immunomodulation Upon STEC Infection of Cattle

In a bovine ligated ileal loop model of STEC infection deploying 1–2 week old calves, 40% of all iIEL, mainly comprising activated CD2^+^ CD3^+^ CD6^+^ CD8α^+^ T cells, were found to be potentially sensitive to Stx1 in that they expressed the receptor for Stx1 [92]. Analysis of iIEL from inoculated loops failed to detect a significant effect of whether the loop was inoculated with a Stx1-producing STEC strain, an isogenic Stx-deficient mutant of that strain or a commensal *E. coli* on proliferative capacity, natural killer cell activity or the cytokine mRNA profile. However, the STEC wild-type strain reduced the percentage of CD8α^+^ T cells in the ileal mucosa compared to loops inoculated with the *stx*-negative mutant [92]. The observation that loop inoculation with STEC led to the reduction of cells capable of binding StxB1 but not of binding anti-CD77 implies that even in vivo CD77^+^ lymphocytes and Stx receptor-positive, Stx-sensitive lymphocytes exist as distinguishable subsets. The shift in iIEL composition was not associated with inhibition of iIEL proliferation in situ, since the majority of IEL from all loops were in the G0/G1 phase of the cell cycle. Because Stxs do not exert a cytotoxic activity for bovine lymphocytes [51,126], the observed effect in vivo may be explained by an interference of Stxs with the recruting of mucosal immune cells. Disturbance of the stromal address code, i.e., the cytokine pattern released by local tissue macrophages [99] as well as iIEL themselves may play a pivotal role here [127]. TGF-β, in particular, induces expression of αEβ7 integrin fostering homing to and retention of IEL in the epithelial layer [128]. Indeed, bovine IEL ex vivo harbour *TGF-β* transcripts and TGF-β protein [82]. However, Stx1 neither affects the number of transcripts nor the amount of protein in vitro, in contrast to the induction of *IL-4* transcription in bovine iIEL by Stx1 in vitro [82].

STEC infections appear to differentially act on the iIEL compartment, i.e., cells interspersed in the epithelial layer, and lymphocytes in organized lymphoid structures in the intestinal wall. Approximately 7% and 10% of jejunal and ileal Peyer’s Patch lymphocytes (PPL) isolated from calves were found to be CD77^+^, respectively, and Stx2 was able to inhibit mitogen-induced proliferation of ileal PPL populations in vitro [52]. Inoculation of ligated bovine intestinal loops with either a Stx2-producing STEC strain or an isogenic *stx*-deficient mutant of that strain significantly reduced CD4^+^ T cells within 24 h in ileal PPL in the presence of Stx2 while the portion of CD8^+^ T cells, CD21^+^ B cells and dendritic cells was unaffected. Expression levels of IFN-α, TNF-α, IL-12, or IFN-γ did not differ between groups [52], suggesting that this aspect of immunomodulation by Stxs occurs independent of a differential cytokine regulation, as also shown for PBMC [55].

However, cattle colonized by STEC O157:H7 after oral infection mount cellular immune responses at intestinal sites characterized by a T_H_1 skew and the reciprocal downregulation of T_Reg_ responses with evidence of strain-specific immunomodulation. Calves challenged with STEC O157:H7 strains showed increased levels mRNA for IFN-γ and the transcription factor T-box expressed in T cells (T-bet) in rectal biopsy specimen as compared to uninfected animals. Expression of both genes peaked at 7 days when animals were challenged with a STEC strain (of phage type 21/28; PT21/28) possessing *stx2a* and *stx2c*, while upregulation was delayed, peaking at 21 days after challenge with a strain possessing *stx2c* (of PT32) only [129]. While pretreatment of bovine colonic explants with TNF-α was shown to upregulate mucin secretion and reduce STEC O157:H7 colonization [130], TNF-α is not induced at the terminal rectum upon experimental STEC O157:H7 infection consistent with the asymptomatic character of STEC infections in cattle [35,131]. Cells isolated from gastrointestinal lymph nodes in this model demonstrated antigen-specific proliferation and IFN-γ release in response to proteins injected into host cells via the LEE-encoded T3SS; however, responsiveness was suppressed in cells isolated from PT32-challenged calves [129]. Lymph node cells showed increased expression of the proliferation marker Ki67 in CD4^+^ T cells from PT21/28-challenged calves, Natural Killer (NK) cells from PT32-challenged calves, and CD8α^+^ and γδT cells from both PT21/28- and PT32-challenged calves following ex vivo restimulation with T3SS proteins [129]. It is interesting that these antigens are able to drive both γδTCR^+^ and CD8α^+^ T cell proliferation, suggesting a role for T cells in the response to colonization and the selection of CD8 T cell clones that are responsive to major histocompatibility complex (MHC) class I-presented STEC O157:H7 peptides, i.e., peptides derived from T3SS effectors [129]. It remains to be determined, though, which of these effects are directly linked to an immunomodulatory activity of Stxs. Transcriptome analyses of recto-anal junction tissue and ileal Peyer’s patches from calves inoculated with *stx*^-^negative *E. coli* O157:H7 also unveiled immunosuppressive effects, the pattern of which differed between primary and secondary infection [132].

When comparing cattle naturally colonized with *E. coli* O157 and shedding the bacteria in high numbers, so called super-shedding (commonly defined as >10^4^ colony-forming units [cfu]/g of faeces), to cattle negative for *E. coli* O157, 351 differentially expressed genes were identified throughout the intestine, with 101 being up-regulated and 250 down-regulated in animals defined as super-shedder at the time of sampling [133]. Concerned genes are involved in increased T cell responses and cholesterol absorption in the distal jejunum and descending colon, and decreased B cell maturation in the distal jejunum of super-shedding cattle [133]. Analysis of gene polymorphisms identified mutations in seven genes involved in leukocyte activation and cholesterol transportation and associated with *E. coli* O157 shedding. At the terminal rectum, 31 genes associated with innate and adaptive immunity were found to be down regulated in super-shedding cattle with 19 genes directly associated with B cell function [134]. Consequently, the ecological niche deployed by STEC when colonizing cattle is not solely a function of bacterial virulence factors including Stxs but also determined by host genetic factors, e.g., those impacting T cell responses and cholesterol metabolism in the intestinal tract.

## 4. Systemic Effects of Shiga Toxins on the Bovine Immune System

Early after the epidemiological link between STEC and human diseases was established and endothelial cells were identified as the prime target for Stxs in man, it was argued that Stxs may also target cells of the hosts’ immune systems. Human IgG- and IgA-producing B cells were shown to be highly susceptible to the cytolethal effect of Stx1 [46,59]. Infections of gnotobiotic piglets with Stx1-producing *E. coli* induce immunosuppression [66]. This lymphotropic effect of Stxs was considered relevant for the pathogenesis of STEC-induced diseases [59], although it seemingly does not prevent generation of Stx-specific antibodies. Stx2e-specific IgG occur in pigs suffering from edema disease [135] and Stx-specific antibodies are present in human sera [136]. Significant IgG responses to LEE proteins (Intimin, Tir, EspA, and EspB) and to O157 LPS develop following experimental STEC O157:H7 challenge of adult beef cattle [137]. Even though responses to Tir and EspA are short lived [137], the latter findings indicate that cattle remain immunologically competent upon STEC infections in general.

### 4.1. Impact on the Humoral Immune Response

Sera and colostrums of cows frequently contain neutralising antibodies against Stx1 and, at lower frequency and with lower titers, against Stx2 [28,138]. This can be taken as evidence that STEC are able to produce Stxs under conditions present in the bovine intestinal tract and, in principle, the toxins are antigenic to the host. Indeed, sera and colostrums of all dams and postcolostral sera of all newborn calves contained Stx1-specific antibodies in a study assessing the quantity and dynamics of maternal and acquired anti-Stx antibodies in a cohort of calves [139]. However, calf serum titers decrease rapidly within the first 6 weeks of age. Despite the fact that all calves had at least one *stx*-positive fecal culture in the first 6 month of life, only five out of 27 calves showed Stx1-specific seroconversion. Sparse Stx2-specific titers were detectable in sera and colostrums of only three dams and in postcolostral sera of their calves and none of the calves developed Stx2-specific seroconversion under conditions of natural exposure. The inability of naturally infected calves to mount a timely anti-Stx response after natural STEC infection was corroborated by a subsequent study, following up 48 calves for 12 month of life [78].

The reason for the significant time delay in mounting bovine anti-Stx responses may be an active immunosuppression by Stxs, an insufficient antigen exposure by small amounts of toxins produced in vivo or a poor immunogenicity of the toxins. The latter may be explained by the structural similarity of StxB with bovine IFNAR [69] and thus the consequence of a centrally induced immunological tolerance. In contradiction to this assumption, a prime/boost i.m. application to calves of Shiga toxoids led to substantial anti-Stx1 as well as anti-Stx2 titres without causing any adverse effects [72]. Vaccination-induced anti-Stx bovine antibodies neutralize the biological activity of the holotoxin in vitro [72] in in vivo [140,141,142] in various models. The poor immunogenicity of Stxs upon natural exposure and the absence of Stx-specific seroconversion seen after experimental STEC infection of calves [29,138], cannot be explained by a damage of B cells by Stxs also. Bovine B cells are similarly sensitive in vitro to Stx1 as CD8α^+^ T cells [51], but Stx2 does not prevent the development of a strong humoral response to O157-LPS in vivo after STEC O157:H7 infection [29,138]. It appears that the B cell compartment in bovines is less sensitive to Stxs in vivo than the T cell compartment. This may particularly apply when B cells are activated by T cell-independent B cell antigens like LPS. Antibodies recognising H7 flagellin are readily detectable in calves colonized with STEC O157:H7 [143]. CD4^+^ T cell epitopes from H7 flagellin were not identified in rectal lymph node CD4^+^ T-cells, though, suggesting that H7 flagellin may act as a T cell-independent antigen. STEC protein antigens do lead to T cell-dependent antibody responses but these responses are generally weak, highly variable, and often short lived [137,144], implying that Stxs rather selectively modulate the response to T cell-dependent antigens via suppression of T cell help [51]. Indeed, serological responses to secreted proteins as well as a co-administered antigen (hen egg lysozyme), were significantly reduced in adult cattle that were immunized with Stx2-containing antigen preparations compared to groups vaccinated with antigens which did not contain the toxin [52]. In turn, protection of newborn calves by administration of Shiga toxoid vaccines resulted in elevated serum levels of H7, H2, and H21 flagellin-specific IgG1 after subsequent natural STEC exposure at 9–12 weeks of age compared to placebo vaccinated calves [145].

### 4.2. Impact on the Cellular Immune Response

Calves inoculated with a *stx*-negative *E. coli* O157:H7 strain, but not those inoculated with a Stx2-positive STEC O157:H7 strain, developed statistically significant lymphoproliferative responses to heat-killed whole antigen derived from the *stx2*-positive strain [29]. Different from pigs showing a general immunosuppression after oral inoculation with a Stx1-producing STEC O111:NM, comprising a depressed mitogenic responsiveness [66], the response to mitogens remains unaffected in STEC inoculated calves. Calves inoculated with the *stx*-negative strain developed an increase in the response to whole antigen over time, which even further increased after challenge with the *stx2*-positive STEC O157:H7 strain, reminescent of a booster reaction. These findings strengthen the assumption that the immunosuppressive effects of STEC infections result from a prevention of the onset of an immune response rather than the downregulation of an established one, in this example induced by the initial inoculation with the *stx*-negative strain. In corroboration of this hypothesis, providing newborn calves with elevated anti-Stx titers by a combination of passive and active immunization prior to acquisition of STEC by natural exposure led to higher responses of peripheral CD4^+^ and CD8α^hi^ memory lymphocytes to re-stimulation with STEC antigens [78]. Cells from vaccinated animals also responded to re-stimulation with antigens from *stx*-negative *E. coli* strains better than non-vaccinated animals implying that vaccination enables calves to build an infection immunity towards antigens shared between STEC and other *E. coli* strains. T cells from vaccinated animals, considered to have been less exposed to Stx, tended to respond more vigorously to mitogens. In light of studies showing that Stx2 impairs the immunogenicity of systemically co-administered antigen also in terms of cellular responses [52] and that STEC colonization affect cattle’s general immune response [143], there is cumulating evidence that Stxs impair immune responses in cattle in a more general manner than is currently appreciated.

## 5. Concept for the Role of Shiga Toxins in STEC Colonization of Cattle

**Figure 2:**
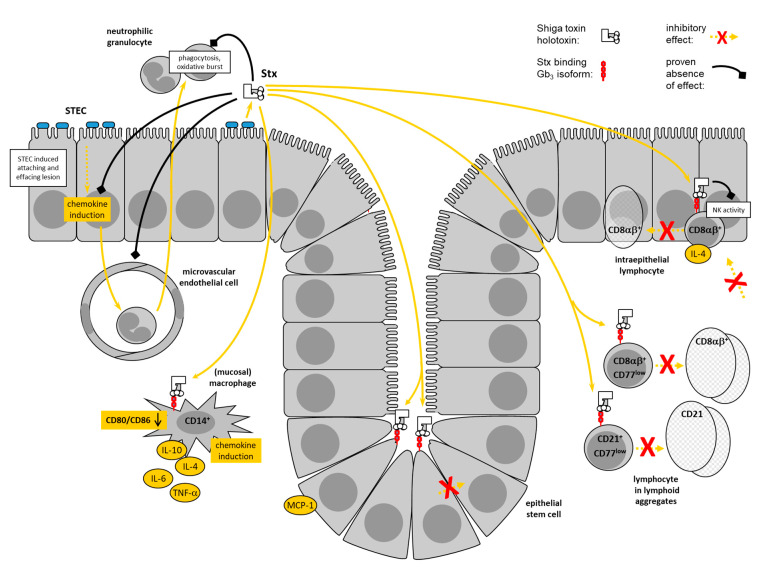
Graphical representation of the intestinal immunomodulation driven by Shiga toxins (Stxs) upon Stx-producing *E. coli* (STEC) infection of cattle. Stx1, but not Stx2, can bind to epithelial cells (EC) at low levels of differentiation whereas differentiated EC, that underwent proliferation and migration to the luminal surface, lacks Stx receptors. Stx1 slightly induces transcription of the gene encoding mono- and lymphocytotropic MCP-1 itself and does not impair the expression of chemokines induced by STEC/EC interactions, partially comprising formation of attaching and effacing lesions. Endothelial cells from bovine large vessels are responsive to Stx but microvascular endothelial cells seem to lack Stx receptors. Bovine neutrophils retain their ability to migrate and perform an oxidative burst in the presence of Stx implying that initial, innate immune responses remain functional during STEC colonization at the bovine intestinal mucosa. Stx2 can restrict epithelial cell proliferation within bovine crypts independent of cell death and apoptosis. The effect might come from direct targeting of EC or indirectly via affecting the release of mediators and growth promotors from adjacent intraepithelial lymphocytes (IEL) and mucosal macrophages. Once Stxs are translocated through the epithelial layer, the latter cell types both respond to Stxs by an altered expression of genes implicated in adaptive immune responses. Moreover, attraction of IEL and macrophages to mucosal sites is disturbed by Stxs. Stxs hinder the transformation of IEL from non-blast cells to blast cells accompanied by an increased transcription of *IL-4* wheras the Natural Killer (NK) cell activity remains unaffected. Once Stx has reached lymphoid aggregates, Stxs block the activation of lymphocytes resulting in a retardation of the development of an effective adaptive immune response. The inability of the host to immediately control STEC colonization results in persistent colonization and shedding of the bacteria (for details and references see text).

STEC only exceptionally causes bloody diarrhea in calves after natural [37] and experimental infections [38,39]. Because STEC infections of adult cattle establish in the absence of intestinal inflammation, it was concluded that STEC have adopted a commensal-like lifestyle in the intestinal milieu in bovines [146]. It needs to be considered, though, that the intestinal mucosa normally exerts a state of physiological inflammation, characterized by the presence of numerous leukocytes in intraepithelial and subepithelial compartments [147]. Bovine STEC isolates possess a number of virulence factors, which enable them to interact with intestinal EC in many different ways [17,31,124,148]. It does not seem plausible that not even one of these factors is recognized by the bovine mucosa as danger signal to alert elements of the innate and adaptive immune system, to escalate inflammation beyond the physiological level, which eventually limits STEC colonization [35,149]. Stx-specific antibodies in cattle [28,138,150] also argue against a principal inability of the local immune system to properly respond to STEC and their products. Consequently, STEC must have developed strategies to actively mitigate the immune response, maintain intestinal homeostasis, and allow for enduring colonization (Figure 2).

The discovery of te Loo et al. [104], that human granulocytes not only bind Stxs via low affinity receptors different from Gb_3_/CD77 but transfer the bound Stx1 to Gb_3_/CD77-expressing endothelial cells, may explain, how strictly enteric STEC infections in man cause extraintestinal complications. Bovine granulocytes, lacking Stx receptors, are unable to transport Stx1 and CD77-positive granulocytes of sheep bind Stx1 with such a high affinity that toxin transfer to sensitive cells is efficiently prevented [151]. However, pigs may develop renal alterations resembling HUS following STEC infections [152] even though porcine granulocytes bind Stx2 via Gb_3_/CD77 receptors [153]. The role of Stx-granulocyte interactions in the course of STEC infections awaits to be fully unveiled, but the significant differences between granulocytes of cattle and sheep shed first light on a presumptive heterogeneity of Stx-cell interactions in different species, equally regarded as asymptomatic STEC carriers and reservoir hosts.

Like their human counterparts, bovine umbilical vein endothelial cells possess Stx receptors [111] and bovine aortic endothelial cells are Stx-sensitive [89]. The absence of such receptors on endothelial cells in the intestinal microvasculature [45] is likely to explain the lack of microangiopathic alterations in the intestine of cattle.

Consequently, the most important target organ for Stxs in cattle is the adaptive branch of the mucosal immune system, proper response of which is disturbed. With mucosal tissue Mø, a target cell type was identified in the bovine intestinal wall, which may act as multiplier of the impact of Stxs in this environment. The cells are present in crypt areas but presumably also attracted to beneath the STEC colonization site at the crypt basis by MCP-1 induced by Stxs in EC. Expression of CD77 on mucosal tissue Mø as well as on bovine monocytes implies, that Stxs interfere with the onset of an immune response even at the level of antigen-specific activation of T cells. This activation may be further mitigated by an increased expression of IL-10 in tissue Mø. An elevated secretion of IL-4 by IEL would drive the differentiation of CD4^+^ T cells in the *Lamina propria* to TGF-β-secreting T_Reg_ cells further preventing the induction of intestinal inflammation following STEC colonization of EC [154,155].

The mucosa overlaying lymphoid follicles in the terminal rectum are the prime colonization site for STEC of serotype O157:H7 in cattle [21,156]. However, STEC O157:H7, in particular in the first days after infection, also colonize the mucosa of ileum, cecum, and colon [19,157]. Non-O157 STEC lack a tropism for the terminal rectum [21,157,158]. Similarities in the expression patterns of surface markers including CD77 of IEL from ileum, colon and cecum (C. Menge and E. A. Dean-Nystrom [National Animal Disease Center, Ames, IA], unpublished) strongly imply that IEL all along the gastrointestinal tract of cattle are sensitive to Stxs. Accordingly, adult cattle poorly develop colonic and rectal mucosal antibodies of the IgG and IgA classes to *E. coli* O157:H7 antigens after repeated infections with Stx2-producing O157:H7 strains followed by subcutaneous injection of purified *E. coli* O157:H7 protein [144].

Beyond the intestinal mucosa, peripheral lymphocytes are affected by Stxs in the early stages of activation in which the cells only express small numbers of Stx receptors, resulting in inhibition of further activation and proliferation and also synthesis of IFN-γ (C. Menge and E. A. Dean-Nystrom, unpublished) and IL-12 [52]. Impeding a developing immune response would be extraordinarily efficient from the point of view of the bacteria because minute amounts of Stxs are sufficient at this stage to block the small number of antigen-specific lymphocytes prior to clonal expansion. Indeed, the delay in mounting a STEC-specific cellular immune response in calves was accompanied by a significantly prolonged shedding of the Stx2-producing O157:H7 challenge strain [29].

Experimental evidence exists that bovine lymphocytes become refractory to Stx1 again once they went through the entire activation process from CD77^low^ to CD77^moderate^ to CD77^high^ stages. However, CD45RO^+^ IEL coexpress CD77 at a considerable percentage (C. Menge and E. A. Dean-Nystrom, unpublished). It cannot be ruled out, therefore, that Stxs even affect the (re)activation of an immunological memory response. No significant local (rectal lymph node cells) and systemic (PBMC) T cell-specific lymphoproliferative responses to LEE proteins developed and no increase in the frequency of antigen-specific IFN-γ producing cells was found in calves experimentally infected with different STEC O157 strains at the age of 8 weeks [145]. All calves did not shed STEC O157 during the 5 weeks immediately prior to challenge [145]. However, it is highly unlikely that the animals did not previously get exposed to LEE antigens, proven to be immunogenic in cattle [159], as calves start shedding STEC strains even in the first weeks of life when raised on a conventional farm [36,139]. Vaccination of cattle with T3SS-secreted LEE proteins reduces STEC O157:H7 shedding [160,161]. Although a three-dose regimen of vaccination resulted in increasing serum titers of antibodies against several T3SS proteins, titers markedly declined 14 days after STEC O157:H7 challenge [161]. Titers against T3SS proteins not sustaining after experimental STEC O157:H7 challenge was repeatedly reported [137]. At least under conditions of experimental STEC infection, therefore, induction or boostering of existing humoral responses to STEC antigens seems to be impaired after STEC infection. If the assumption holds that Stxs even affect memory responses, a delayed reactivation of the vaccine-induced memory response upon STEC infection would restrict the protective effects of such vaccines to the period in which the induced effector mechanisms are still in an elevated activity state [161] in which they are less sensitive to the immunomodulatory effect of Stx.

Most studies aiming at determining quantities and duration of STEC shedding by cattle did not investigate adaptive immune responses and followed up long-term colonization beyond five weeks after experimental inoculation [162]. When assessing long-term colonization capabilities of different pathovars in sheep, Cornick et al. observed that STEC O157:H7 strains tended to persist more frequently than did strains of other pathovars after experimental infection [163]. The absence of *stx2* or the entire *stx2*-encoding phage in isogenic mutants of one of the STEC O157:H7 strains did not significantly impact on the magnitude or duration of *E. coli* O157:H7 shedding [164]. Under field conditions, however, Wang et al. observed the potential acquisition of the phage-encoded *stx2* gene by a bovine *E. coli* isolate of serotype O22:H8 in a closed Canadian beef herd [165]. While the majority of such events appear to be transient in nature, carriage of *stx2* in O22:H8 persisted and strains carrying *stx2* became the dominant O22:H8 genotype in the cattle cohort sampled one year later [165]. This argues in favor of a selective advantage of *stx*-phage carriage for the respective *E. coli* strain in the long term. This may not always become apparent in comparably short-term experimental infection studies, in particular when considering that rather high numbers of inoculating bacteria are normally administered which may, by several orders of magnitude, exceed the minute infection doses required to infect calves [18].

## 6. Presumptive Drivers of STEC Preservation and Evolution in Cattle

In a simplistic view, possession of the *stx* gene would confer a fitness benefit in the bovine intestinal habitat at least for those STEC strains having acquired the ability to engange with the epithelial layer, like LEE-positive STEC and EAEC/EHEC hybrid strains. Given that a plethora of different STEC strains are present in bovines, including LEE-negative strains and EAEC strains [36,124,139,157,166], the question arises why potential advantages of *stx*-converting phage carriage sufficiently compensate for the metabolic burden and the, potentially detrimental, cytolytic effects of *stx*-phages. Beyond promoting STEC colonization by immunomodulation exerted by Stx, other traits fostering cattle-STEC coevolution are considered, some encoded by or functionally linked to the genome of *stx*-converting phages.

### 6.1. Acquisition of Other Virulence-Associated Genes

With the advent of whole genome sequencing technology, it became evident that the STEC pathovar has arisen multiple times by independent acquisition of virulence factors on mobile genetic elements [167,168]. These horizontally acquired regions add over 1Mb of extra genetic information to the “core” genome present in non-pathogenic *E. coli* K-12 and encode colonization factors including the T3SS, associated secreted effector proteins, fimbriae, and Stxs [168,169,170]. Some EHEC O157 and O26 genes were confirmed by mutagenetic approaches to be important for bacterial colonization in cattle [33,34]. Preexisting [171] or vaccine-induced [172] antibodies against *E. coli* O157:H7 Intimin gamma partially reduce fecal *E. coli* O157:H7 shedding in calves following experimental infection. Immunization of calves with recombinant T3SS-associated proteins EspA, Intimin, and Tir from STEC O157:H7 significantly reduce shedding of STEC O157 from experimentally colonized calves, and protection could be augmented by the addition of H7 flagellin to the vaccine formulation [173].

However, bovine STEC strains are not all equally able to colonize cattle [124]. Comparison of persistent colonizing STEC (STEC^per^; shedding for >4 months) and sporadically colonizing STEC (STEC^spo^; shedding for <2 months) revealed that STEC^per^ isolates with heterogeneous phylogenetic backgrounds are clustered together by their accessory genome, separating them from the STEC^spo^ isolates. STEC^per^ possess more often genes encoding factors involved in adherence (*eae*, *efa-1*/*lifA* (EHEC factor for adherence), *lpfA*, *tccP*), T3SS effector proteins (*espB*, *espJ*), non-LEE encoded effectors (*nleA*, *nleB*, *nleC*), and *stx1*. In contrast, STEC^spo^ isolates encoded more often Stx2 or ToxB, a protein involved in adherence. While possession of *efa1* was more likely to be associated with a persistent than a sporadic phenotype in this study [124], *efa1* was not found in any of the persistent serotypes identified in another study [165], implying that *efa1* is not sufficient as biomarker for STEC^per^.

Most EPEC and non-O157 STEC strains express lymphostatin (also referred to as LifA, Efa-1, the gene product of *efa-1*/*lifA*), a chromosomally encoded 365-kDa protein which inhibits proliferation of human [70,71,174] and murine lymphocytes [175]. Lymphostatin is a putative glycosyltransferase implicated in intestinal colonization of cattle by STEC serogroup O5, O111 [120], and O26 strains [176] early after infection, before adaptive responses may be expected to have developed [177]. Lymphostatin inhibits mitogen-activated proliferation of bovine T cells and, to a lesser extent, proliferation of cytokine-stimulated B cells, but not NK cells [178]. It broadly affects the T cell compartment, inhibiting all cell subsets (CD4, CD8, WC-1, and γδT cells) and several cytokines (IL-2, IL-4, IL-10, IL-17A, and IFN-γ) and renders T cells refractory to mitogen. An amino acid substitution that ablated lymophostatin activity against bovine PBMC [176] and T cells [179] did not significantly attenuate a STEC O26:H^−^ strain in a calf intestinal colonization model, indicating that the immunomodulatory role of LifA may not be strictly necessary for persistence of STEC in cattle [176] and sheep [119].

Intimins not only represent important effector molecules for the colonization of intestinal epithelia by STEC but also costimulate mucosal immune cells and induce a strong intestinal inflammation in mice [180]. Bovine immune cells, however, resist a proinflammatory effect of the Intimins. The C-terminal 280 amino acid portions of the Intimin subtypes beta and gamma do not bind to bovine PBMC and iIEL, nor do they influence the proliferative capacity of the cells or expression of various cytokine genes (*IL-2*, *IL-4*, *IL-8*, *IL-10*, *IFN-γ*, and *TNF-α*) [181].

In general terms, cattle-STEC coevolution has led to the accumulation of virulence factors in the STEC genome but virulence gene patterns are not strictly conserved among strains, in particular when comparing strains exhibiting different levels of adaptation to cattle. Although the role of adhesins in colonization is undisputed, adhesins exerting immunomodulatory effects in other hosts do not act the same way in cattle. Stxs stand out as the only immunomodulating virulence factor shared, by definition, by all STEC strains.

### 6.2. Differential Regulation of Virulence Genes, Acquisition, and Loss of Metabolic Traits

Numerous attempts have been undertaken to subdivide the many different STEC strains that are shed by cattle in order to predict a given strain’s degree of threat to human health. Various levels of host adaptation could be traced back to certain expression patterns of virulence genes. STEC O157:H7 strains, e.g., were found to express *iha*, *espA*, *rfbE*, and *ehxA* to different extents upon natural infections of humans and cattle [182]. Spontaneous Stx production is higher in HUS-associated EHEC clones than in bovine STEC isolates and Stx1 production is induced more strongly by iron deprivation in vitro in the former [183]. A lower capacity to produce Stx2 in bovine STEC correlates with the presence of the Q_21_ allele of the late antiterminator Q upstream of *stx* in the genome of *stx*-converting prophages, whereas strongly inducible Stx production seems to be linked to the Q_933_ allele [184]. Indeed, a support vector machine analysis of bovine *E. coli* O157 isolate sequences, by comparison with sequences from human isolates, identified cattle strains posing a superior threat to human health [185]. Distinction was possible despite the fact that the majority of the isolates considered were members of previously defined pathogenic lineages and encoded key virulence factors. The major differences between human and bovine *E. coli* O157 isolates were the relative abundances of predicted prophage proteins.

Phage type (PT) 21/28 strains of serotype O157:H7, particularly associated with super-shedding events [186], have a lower median level of T3SS expression compared to other strains [187]. Deletion of *stx2*-converting phages increases the level of T3SS expression [187]. The presence of an integrated *stx2*-converting phage represses LEE-encoded regulator (Ler) induction of the LEE. This regulation involves the CII phage regulator and can be relieved by ectopic expression of a cognate CI regulator. A complex regulatory network senses and responds to a myriad of host- and microbiota-derived signals in the infected gut to control transcription of the LEE. These signals include microbiota-liberated sugars and metabolites in the gut lumen, molecular oxygen at the gut epithelium, and host hormones (reviewed in [188]). AE pathogens also recognize physical signals, such as attachment to the epithelium, and the act of effector translocation remodels gene expression in infecting bacteria. QseF of the bacterial two-component system (TCS) QseEF promotes the expression of Stx2 [189,190] and the T3SS effector TccP/EspFU, which is necessary for AE lesion formation in STEC [191]. Importantly, QseB of the cooporating TCS QseCB (directly) and QseF (indirectly) also repress the FusKR TCS, alleviating fucose-mediated repression and thus shifting the regulatory balance toward the expression of the entire LEE [192]. The data imply that *stx*-converting bacteriophages are an integral part of a network to regulate T3SS and thereby to co-ordinate epithelial cell colonization.

Carriage of *stx*-phages even profoundly affects *E. coli* gene expression relevant for carbon source utilization [193]. *Stx*-phage lysogeny in *E. coli* K-12 leads to the differential expression of more than 150 bacterial host genes and enhances its acid resistance and motility [194]. This effect is also mediated by the phage-encoded regulator CII [195]. The phage-encoded anti-repressor Cro, which mediates the switch to the phage lytic cycle, not only activates LEE gene expression, but affects the expression of nearly 900 genes [196] including upregulation of genes involved in mixed acid fermentation, while genes encoding NADH dehydrogenase I, TCA cycle enzymes, and proteins involved in the transport and assimilation of carbon sources are downregulated [193]. Stx2a phage lysogens show a significant decrease in the cell respiration with gluconeogenic substrates such as amino acids, nucleosides, carboxylic and dicarboxylic acids with prophage-encoded factors distinct from CI and Cro being responsible for these phenotypes [193].

Metabolic adaptation to the ecological niche in bovines may particularly involve genomic regions contributing to growth in bovine gastrointestinal mucus [167]. Possession and differential regulation of metabolic traits impact on the colonization phenotype of STEC strains in cattle [197]. STEC^spo^ strains produce significantly more biofilm than STEC^per^ at lower temperatures. Glyoxylic acid and L-rhamnose are metabolized by STEC^spo^, but not or only by some STEC^per^. Genomic sequences of the respective *glc* and *rha* operons contain mutations and frameshifts in uptake or regulatory genes, particularly in STEC^per^ [197]. While STEC^spo^ conserved features leverage survival in the environment, acquisition of a persistent colonization phenotype in the cattle reservoir is accompanied by the loss of metabolic properties and genomic mutations in the underlying genetic pathways.

### 6.3. Exchange of Immunodominant Surface Structures

O-antigens as part of the LPS, a major component of the outer membrane of gram-negative bacteria, are instrumental for host colonization and bacterial niche adaptation [198]. O-antigens are highly immunogenic and, therefore, targeted by the adaptive immune system. Due to this strong selective pressure, the O-antigen is one of the most variable bacterial cell constituents, with variation in the types of sugars present, their arrangement within the O-unit, and the linkages between O-units [199,200]. Although seemingly a rare event, evidence exists that cattle-STEC co-evolution results in modification of the bacterial cell surface to enable STEC strains to escape from the host response. Whole genome sequence analysis of STEC^per^ revealed that STEC isolates belonging to serotypes O156:H25 and O182:H25 fell into two multilocus sequence types differing only by a single nucleotide [201]. The fitness- and virulence-associated genome regions including the integration sites and sequences of the *stx*-converting bacteriophages were almost indistinguishable but the O-antigen gene cluster (O-AGC) regions were fundamentally different, specific for the respective serotype. Epidemiological and molecular data suggests a recent switch of the O-AGC between STEC strains colonizing cattle with O156:H8 strains having served as DNA donors. This incident may serve as further evidence that bovine STEC are subject to evolutionary pressure exerted by the host immune system.

### 6.4. Antiviral Activity of Shiga Toxins

Stx1 [126] and Stx2 [202] have antiviral properties for bovine viruses as assessed by the impact of Stxs on PBMC from cows infected with bovine leukemia virus (BLV) [126]. PBMC from BLV-positive animals display spontaneous lymphocyte proliferation in vitro, which is strongly inhibited by Stx1. This antiviral activity is exerted by the catalytically active A subunit and does not require toxin uptake by the cells via the classical receptor-mediated route [202]. Instead, StxA1 targets highly permeable virus-expressing cells. The effect is not restricted to BLV but StxA1 also prevents replication of bovine immunodeficiency virus (BIV) in infected cells in a concentration-dependent manner [203]. The complex retrovirus BIV is even more sensitive than the single-stranded RNA virus BLV [204]. Antiviral activities of Stxs seem to have clinical implications as intestinal STEC were shown to mitigate BLV infection in experimentally infected sheep [205]: high level STEC carriage correlated with good health, whereas poor weight gain, distress, and tumor development occurred only among animals with low STEC scores [206].

### 6.5. Anti-Protozoal Activity of Shiga Toxins

It was suggested that some of the virulence factors of STEC O157:H7 enhance the survival of these bacteria and contribute to the evolution of their virulence to colonize humans by providing protection against predation by bactivorous protozoa, nematodes or other predators in the soil, water or the gastrointestinal tract of their bovine hosts. Indeed, carriage of a *stx*-converting prophage of STEC O157:H7 increased the rate of survival of *E. coli* bacteria in the food vacuoles of *Tetrahymena* spp. in some studies [207]. Others found no evidence of a protective effect of either Stxs or the products of other bacteriophage genes on protozoan predation of *E. coli* [208]. A transcriptome analysis of STEC O157: H7 revealed that protection from oxidative stress by bacterial factors other than Stx and not associated with *stx*-converting phages play a major role in the resistance of the bacteria from predation by *Tetrahymena* [209]. In a *Acanthamoeba castellani* model, Stx-dependent and independent cytotoxic effects to the co-cultured amoebae impaired STEC survival [210]. The contribution of Stxs to bacterial survival when facing protozoa, therefore, seems to be variable depending on the conditions of the challenge, and the protozoan models used in the studies.

### 6.6. Horizontal Transfer of STEC and Super-Shedding Events in Cattle Herds

In periods of low pathogen exposure (on pasture), STEC shedding rates may temporarily drop [26], but the same STEC clone can be maintained in a single herd for several months and years [17,27]. A previous STEC infection does not protect from re-infection even with the same strain [24,29]. Super-shedding of STEC by cattle seems to be the major source of deposition into the environment and has an important role in augmenting cattle-to-cattle transmission [211]. Such super-shedding is not confined to certain animals [212] but viewed as an occasional phenomenon with the potential to occur in any individual bovine.

It appears that the super-shedding events are more determined by factors expressed by certain STEC strains than by host factors. In the United Kingdom, emergence of phage type (PT) 21/28 O157 was linked to super-shedding [80]. Although encoding for Stx2a and Stx2c, Stx2a is the main toxin produced by these strains. Stx induction is associated with more rapid induction of regulatory gene expression from the *stx2a*-prophage compared to that from the *stx2c*-prophage. Stx2a expression increased animal-to-animal transmission and the number of sentinel animals that became super-shedders in these studies. *Stx2a*-enhanced transmission seems to occur independent of suppression of *E. coli* O157-specific immune responses at the site of colonization but restriction of epithelial regeneration by Stx2a was identified as the principal factor fostering shedding and transmission [80]. However, vaccination with a receptor and porin proteins-based vaccine but not a direct-fed microbial (*Lactobacillus acidophilus*-based) reduced the prevalence of *E. coli* O157:H7 high shedders commercial feedlot cattle [213] pointing to some level of control of super-shedding events by the host. Understanding the underlying host–pathogen interactions are of utmost importance as the super-shedding events, though relatively rare [214], contribute significantly to human risk [215].

### 6.7. Vertical Transfer of STEC in Cattle Herds

It would be plausible that super-shedding events and concomittent animal-to-animal spread also is determinative for the infection rate of young cattle at the time they are exposed to and become infected by one or several STEC strains for the first time in life. It was speculated that dams shed more STEC during and after calving, because pregnancy and calving would be expected to be a stressor for the cows. However, calving and the number of animals shedding STEC do not correlate [216]. Several studies indicate that some restrictions apply for the transmission of STEC from dams to their offspring. Shaw et al. showed that STEC strains isolated during 21 weeks after birth from calves and their dams differed with respect to the presence of *stx2* and the *eae* gene [36]. No calf shed the same STEC serogroup (excluding O?) as its dam at birth or at the end of the study. The most frequently detected serogroups in calves were serogroup O26 and provisional serogroup E40874, whereas in dams, serogroup O91 and provisional serogroup E54071 dominated. STEC O26 shedding appeared to be associated with very young calves and declined as the calves aged, whereas STEC O2 shedding was associated with housing of the animals. STEC O26 strains from calves were characterized by the presence of the *stx1*, *eae*, and *ehxA* genes, whereas *stx2* was associated with STEC O2 and provisional serogroup E40874. Similarly, all calves studied for more than 9 weeks of life shed STEC on at least one occasion in another study [139]. The portion of STEC-shedding calves constantly rose until weaning, in week 12, and remained constantly high thereafter. There is cumulating evidence, therefore, that STEC strains possessing *stx1* are better equipped to colonize suckling calves, while *stx2*-possessing strains dominate in weaned calves and adult cattle [36,139,216]. It must be considered, therefore, that vertical transmission of STEC is less important for infections of calves at young ages than transmission between animals of the same age and, under most circumstances of modern husbandry, sharing the same pen. It remains to be determined how the specific characteristics of strains circulating in calf cohorts correlate with features of STEC strains particularly adapted to persistently colonize adult cattle.

## 7. Options for Countermeasures to the Immunomodulatory Effects of Shiga Toxins in Cattle

Up until now, the success of attempts to vaccinate cattle was mostly restricted to single subpopulations of STEC, e.g., O157 strains [160] harbouring the genes for Tir (translocated intimin receptor) [160], for adhesion factor Intimin [217], for Esps (*E. coli* secreted proteins) A and B [137,218], or for flagellin H7 [219]. By definition, Stxs are the only virulence factors harboured by all STEC strains. Stxs act as immunomodulating agents during bovine STEC infections [29,51,82,92,99] by affecting the early phases of immune activation [50,63]. Consequently, Stxs may be most effective upon first STEC infection of hitherto immunologically naïve animals at the time they first encounter STEC antigens. Calves become infected with many different STEC strains in their first weeks of life [36,139]. In the absence of Stxs, animals may be able to mount an efficient adaptive immune response with the potential to prevent persistent STEC colonization of the intestinal mucosa. However, Stxs always co-occur with STEC antigens in spatial and temporal terms during infection. In this particular situation, Stxs apparently hinder calves from properly responding, creating an immunologically privileged niche and thereby paving the way for persistent colonization. Intervention measures aiming at interfering with the detrimental effect of Stxs on the immune response developing after natural infection must be effective, therefore, prior to first STEC encounter by the animals.

Bovine lactoferrin (bLF) was shown to degrade StxB2 and to partially protect mammalian cells from cytotoxic effects exerted in co-cultures with STEC bacteria [220]. bLF is internalized by bovine rectal epithelial cells and becomes translocated to the nuclei, in particular when cells are co-cultured with *E. coli* O157:H7 [221]. bLF mitigates shedding of *E. coli* O157:H7 by cattle [222], but only if applied rectally, i.e., at the principal colonization site of bacteria of this serotype [222]. Treatment approaches such as monoclonal antibodies or antisera directed against Stxs and toxin receptor analogs, developed for the treatment of human STEC infections [16] have not been tested for their efficacy to protect cattle against the immunmodulatory effects of Stxs but will likely remain too expensive to be considered for such use.

Immunization of pregnant cows with Stxs results in colostrums enriched in anti-Stx1 and anti-Stx2 [142,223]. Such colostrums, or preparations thereof, reduce STEC shedding by experimentally infected dogs [140] and mitigate Stx2 absorption from the intestine of mice [141]. They protect weaned mice from a lethal dose of STEC O157:H7, avoid the inhibition of water absorption induced by *E. coli* O157:H7 in human colon fragments, and mitigate the pathogenicity of STEC O157:H7 in rat colon loops, although some of these effects were to be partially attributed to bLF in the preparations [142]. First infections of animals at calf’s age coincide with the lack of Stx-specific antibodies. Development of anti-Stx antibodies is remarkably delayed after natural [139] and experimental STEC infection of cattle [138]. Application of Stx vaccines early in life may protect from the immunomodulatory effects of Stxs and concomittendly enable calves to actively mount a primary immune response to antigens other than Stxs that are harboured by STEC strains circulating in the respective cohort.

When using murine antibody 13C4, specific for StxB1, as a model for Stx1-specific immunoglobulins, it became apparent that pre-incubation with Stx1 holotoxin at molar excess of the antibody fully blocked binding and biological effects of Stx1 to bovine lymphocytes [63]. By contrast, pre-incubation of StxB1 with lower than equimolar amounts of the antibody enhanced binding of StxB1 to the cell surface and the antibody blocked internalization only if binding occured after StxB1 had bound to its receptor [63]. Moreover, addition of Stx2B to a vaccine formulation consisting of LEE-encoded proteins did not result in a superior level of protection in terms of reduced STEC O157:H7 shedding compared to vaccination with the LEE-encoded proteins only [224]. Apparently, protection of cattle against the immunomodulatory effects of Stxs requires vaccines containing antigens derived from all subunits of the toxins without being immunomodulatory on their own.

Chemically inactivated Stx2e holotoxin was only partially effective in protecting piglets against oedema disease [225]. A more promising approach is the inactivation of Stx by genetic modification. Replacement of amino acids E167 and R170, located within the enzymatically active cleft of Stx2e [226,227] and vaccination with the recombinant protein fully protects piglets against challenge with native Stx2e [228]. Similar results have been reported for mice [229,230]. Recombinant Shiga toxoids (rStx1_mut_ and rStx2_mut_), in both of which E167 and R170 were replaced with glutamine (Q) and leucine (L), respectively, neither induce transcription of *IL-4* mRNA in bovine iIEL cultures nor reduce the expression of Gb_3_/CD77 on B and T cells from peripheral blood and of CD14 on monocyte-derived Mø [72]. Antibodies in sera of cattle naturally infected with STEC recognize rStx_mut_ toxoids equally well as the wild type toxins. Immunisation of calves with rStx1_mut_ plus rStx2_mut_ induces antibodies neutralizing Stx1 and Stx2. Consecutively, these vaccine candidates were applied in a calf cohort for passive (colostrum from immunized cows) and active (intra-muscularly at 5th and 8th week) vaccination [78]. Stx-neutralizing antibodies were effectively transferred from dams to calves via colostrum and serum antibody titers in calves differed significantly between the vaccinated and the control group until the 16th week of life. In the 11th and 16th week vaccinated calves were able to actively build significantly higher anti-H7 IgG1 levels than control animals. OD values for anti-H7 IgG1 detection correlated positively with anti-Stx1 titers [145]. CD4^+^ and CD8^+^ T memory cells from vaccinated animals responded more pronounced than those of control calves to lysates of STEC and *E. coli* strains isolated on the farm. Summarized for the entire observation period of one year after birth, less fecal samples from vaccinated calves were *stx1* and/or *stx2* positive. Even though Stx-specific antibodies directed against StxB may exert adverse effects, transfer to and induction in young calves of Stx-neutralizing antibodies by Shiga holotoxoid vaccination proved to be an effective measure to protect calves from the detrimental effects of Stxs on the developing immune response and subsequent persistent colonization. To be considered alternatively to generating recombinantly expressed toxoids for vaccine production, a recent study showed that pre-inoculation with an avirulent K88 (F4)+ enterotoxic *E. coli* (ETEC) strain expressing adhesive fimbriae and a non-toxic form of LT confers significant short term protection from challenge with a virulent ETEC strain that expresses the same fimbrial adhesion and enterotoxin [231].

## 8. Conclusive Summary

Despite numerous efforts to establish suitable animal models, none of them entirely replicate the EHEC infection and HUS observed in humans patients [232]. The principal reasons for this likely are species differences in the distribution of functional Stx receptors on cells and in tissues and the different cellular responses of receptor-bearing cells. Even bovines, not developing any known clinical or even histopathological signs upon persistent colonization by STEC that could be ascribed to the effect of Stxs, possess a peculiar Stx receptor distribution [8]. Cumulating evidence, reviewed herein, suggests that Stx receptor expressing bovine cells are also capable of responding to Stxs. In stark contrast to several sensitive cell types in humans, the effects of Stxs on bovine cells are of modulating rather than cytolethal nature. Therefore, it seems to be impossible to easily distuinguish Stx-sensitive from Stx-resilient species per se. Because several studies provided evidence for Stx-related effects occurring in vivo, production and secretion of Stxs must be regarded a pathogenic feature of STEC upon colonization of bovines. The specific distribution pattern of Stx receptors in cattle may not just explain why the animals do not develop Stx-related organ damage but also implies that acquisition of *stx*-converting phages by certain *E. coli* clones was an evolutionary step towards adaptation to a reservoir host.

The prevalence of EHEC serovars (e.g., O157:H7, O26:H11) is significantly lower in cattle compared to other STEC serovars [233]. Even within the O157:H7 serovar only a small subset of bovine isolates has the potential to cause diseases in humans [185]. Host-specific (niche adaptation) information is stored in the accessory genome [185], consistent with findings that STEC strains colonizing the gastrointestinal tract of cattle persistently share similar sets of accessory genes [124,165]. In turn, pre-challenge with a STEC O22:H8 strain, a serotype which has been associated with several habitats [165] but rather rarely associated with human illness, significantly reduces shedding of STEC O157:H7 by calves [234]. STEC O22:H8 did not inhibit growth of STEC O157:H7 in vitro, suggesting that strains with different levels of host adaptation may compete with more virulent strains in certain niches like at the bovine recto-anal junction [234]. The partial elucidation of the genetic patterns determining host adaptation has important implications for development of effective control strategies [235] and risk assessments but are seemingly too complex and variable [165] to be deployed for creating defined and targeted countermeasures like vaccines.

Being part of prophage genomes, *stx*-encoding genes are subject to frequent recombination events [10]. It is assumed, therefore, that novel STEC strains exerting an elevated human pathogenic potential are continuously formed in the bovine interstinal tract [236]. Calves become infected with a plethora of different STEC strains early in life via horizontal or vertical transmission and may shed these bacteria for several months and shed STEC quantities may be considerably high at some sampling points [17,139,237,238]. To prevent humans from EHEC infection, interventions must be applied at several stages of the food chain, starting at animal and herd level [239,240] and continuing in slaughterhouses, meat and milk processing plants [241], dairies, distributors, and households [242]. A systematic review of vaccinations to reduce the shedding of *E. coli* O157 in the faeces of domestic ruminants revealed that vaccination may be a sensible control option [243]. Current vaccination strategies are promising but only succeed partially in reducing *E. coli* O157:H7 excretion [161,242,244]. In some instances, e.g., when vaccinating cattle against H7 flagellin, an important adhesion factor to bovine intestinal epithelium during early stages of colonization [245], systemically induced H7-specific IgG may even impair innate immune responses to *E. coli* O157:H7 when getting into contact with the epithelium via neutralisation of TLR5-mediated activation of epithelial cells [242].

The highly virulent O104:H4 EAEC/EHEC hybrid strain having caused the large HUS outbreak in 2011 possesses a blended virulence profile of human adapted EAEC and STEC, adapted to ruminant hosts. The emergence of such a virulent strain challenged the established model of STEC epidemiology. When interacting with jejunal and colonic epithelial cells of human and bovine origin, the O104:H4 strain appeared to be adapted to humans, based on adhesion, virulence factor expression and immune modulation. However, after experimental infection of calves, the strain was recovered until 24 days post infection, implying that cattle can also carry unusual STEC strains at least transiently.

Stx, the principal STEC virulence factor, modulates immune responses in cattle and Shiga toxoid vaccination reduced the incidence of *stx*-positive fecal samples in a calf cohort. There is cumulating evidence, therefore, that the ability to produce Stxs is sufficient to qualify pathogenic *E. coli* strains for both, being successful colonizers in ruminants as well as emerging human pathogens. The development of effective interventions strategies to prevent human EHEC infections must target, therefore, all STEC strains present in cattle populations with the immunomodulatory Stxs as prime target structure for intervention strategies to be effective at the level of the natural host species.

## References

[1] Hajishengallis G., Nawar H., Tapping R.I., Russell M.W., Connell T.D. (2004). The Type II heat-labile enterotoxins LT-IIa and LT-IIb and their respective B pentamers differentially induce and regulate cytokine production in human monocytic cells. Infect. Immun..

[2] Simmons C.P., Ghaem-Magami M., Petrovska L., Lopes L., Chain B.M., Williams N.A., Dougan G. (2001). Immunomodulation using bacterial enterotoxins. Scand. J. Immunol..

[3] Hajishengallis G., Tapping R.I., Martin M.H., Nawar H., Lyle E.A., Russell M.W., Connell T.D. (2005). Toll-like receptor 2 mediates cellular activation by the B subunits of type II heat-labile enterotoxins. Infect. Immun..

[4] Heim J.B., Hodnik V., Heggelund J.E., Anderluh G., Krengel U. (2019). Crystal structures of cholera toxin in complex with fucosylated receptors point to importance of secondary binding site. Sci. Rep..

[5] Sixma T.K., Stein P.E., Hol W.G., Read R.J. (1993). Comparison of the B-pentamers of heat-labile enterotoxin and verotoxin-1: Two structures with remarkable similarity and dissimilarity. Biochemistry.

[6] Karmali M.A. (1989). Infection by verocytotoxin-producing *Escherichia coli*. Clin. Microbiol. Rev..

[7] Scheutz F., Teel L.D., Beutin L., Pierard D., Buvens G., Karch H., Mellmann A., Caprioli A., Tozzoli R., Morabito S. (2012). Multicenter evaluation of a sequence-based protocol for subtyping Shiga toxins and standardizing Stx nomenclature. J. Clin. Microbiol..

[8] Menge C. (2020). Molecular Biology of *Escherichia coli* Shiga Toxins’ Effects on Mammalian Cells. Toxins.

[9] Gannon V.P., Gyles C.L. (1990). Characteristics of the Shiga-like toxin produced by *Escherichia coli* associated with porcine edema disease. Vet. Microbiol..

[10] Schmidt H. (2001). Shiga-toxin-converting bacteriophages. Res. Microbiol..

[11] Herold S., Karch H., Schmidt H. (2004). Shiga toxin-encoding bacteriophages—Genomes in motion. Int. J. Med. Microbiol..

[12] O’Brien A.D., Holmes R.K. (1987). Shiga and Shiga-like toxins. Microbiol. Rev..

[13] Ohmura M., Yamamoto M., Tomiyama-Miyaji C., Yuki Y., Takeda Y., Kiyono H. (2005). Nontoxic Shiga toxin derivatives from *Escherichia coli* possess adjuvant activity for the augmentation of antigen-specific immune responses via dendritic cell activation. Infect. Immun..

[14] Lingwood C.A. (1996). Role of verotoxin receptors in pathogenesis. Trends Microbiol..

[15] Moxley R.A. (2004). *Escherichia coli* 0157:H7: An update on intestinal colonization and virulence mechanisms. Anim. Health Res. Rev..

[16] Muhlen S., Dersch P. (2020). Treatment Strategies for Infections With Shiga Toxin-Producing *Escherichia coli*. Front. Cell. Infect. Microbiol..

[17] Geue L., Segura-Alvarez M., Conraths F.J., Kuczius T., Bockemuhl J., Karch H., Gallien P. (2002). A long-term study on the prevalence of shiga toxin-producing *Escherichia coli* (STEC) on four German cattle farms. Epidemiol. Infect..

[18] Besser T.E., Richards B.L., Rice D.H., Hancock D.D. (2001). *Escherichia coli* O157:H7 infection of calves: Infectious dose and direct contact transmission. Epidemiol. Infect..

[19] Grauke L.J., Kudva I.T., Yoon J.W., Hunt C.W., Williams C.J., Hovde C.J. (2002). Gastrointestinal tract location of *Escherichia coli* O157:H7 in ruminants. Appl. Environ. Microbiol..

[20] Girard F., Dziva F., van Diemen P., Phillips A.D., Stevens M.P., Frankel G. (2007). Adherence of enterohemorrhagic *Escherichia coli* O157, O26, and O111 strains to bovine intestinal explants ex vivo. Appl. Environ. Microbiol..

[21] Naylor S.W., Low J.C., Besser T.E., Mahajan A., Gunn G.J., Pearce M.C., McKendrick I.J., Smith D.G., Gally D.L. (2003). Lymphoid follicle-dense mucosa at the terminal rectum is the principal site of colonization of enterohemorrhagic *Escherichia coli* O157:H7 in the bovine host. Infect. Immun..

[22] Pohlenz J.F., Dean-Nystrom E.A. (2004). Colonisation of *Escherichia coli* O157:H7 on squamous epithelial cells at the rectal-anal junction. Vet. Rec..

[23] Stoffregen W.C., Pohlenz J.F., Dean-Nystrom E.A. (2004). *Escherichia coli* O157:H7 in the gallbladders of experimentally infected calves. J. Vet. Diagn. Investig..

[24] Cray W.C., Moon H.W. (1995). Experimental infection of calves and adult cattle with *Escherichia coli* O157:H7. Appl. Environ. Microbiol..

[25] Dean-Nystrom E.A., Stoffregen W.C., Bosworth B.T., Moon H.W., Pohlenz J.F. (2008). Early attachment sites for Shiga-toxigenic *Escherichia coli* O157:H7 in experimentally inoculated weaned calves. Appl. Environ. Microbiol..

[26] Jonsson M.E., Aspan A., Eriksson E., Vagsholm I. (2001). Persistence of verocytotoxin-producing *Escherichia coli* O157:H7 in calves kept on pasture and in calves kept indoors during the summer months in a Swedish dairy herd. Int. J. Food Microbiol..

[27] Liebana E., Smith R.P., Batchelor M., McLaren I., Cassar C., Clifton-Hadley F.A., Paiba G.A. (2005). Persistence of *Escherichia coli* O157 isolates on bovine farms in England and Wales. J. Clin. Microbiol..

[28] Pirro F., Wieler L.H., Failing K., Bauerfeind R., Baljer G. (1995). Neutralizing antibodies against Shiga-like toxins from *Escherichia coli* in colostra and sera of cattle. Vet. Microbiol..

[29] Hoffman M.A., Menge C., Casey T.A., Laegreid W., Bosworth B.T., Dean-Nystrom E.A. (2006). Bovine immune response to shiga-toxigenic *Escherichia coli* O157:H7. Clin. Vaccine Immunol..

[30] Magnuson B.A., Davis M., Hubele S., Austin P.R., Kudva I.T., Williams C.J., Hunt C.W., Hovde C.J. (2000). Ruminant gastrointestinal cell proliferation and clearance of *Escherichia coli* O157:H7. Infect. Immun..

[31] Dean-Nystrom E.A., Bosworth B.T., Moon H.W., O’Brien A.D. (1998). *Escherichia coli* O157:H7 requires intimin for enteropathogenicity in calves. Infect. Immun..

[32] Cornick N.A., Booher S.L., Moon H.W. (2002). Intimin facilitates colonization by *Escherichia coli* O157:H7 in adult ruminants. Infect. Immun..

[33] Dziva F., van Diemen P.M., Stevens M.P., Smith A.J., Wallis T.S. (2004). Identification of *Escherichia coli* O157: H7 genes influencing colonization of the bovine gastrointestinal tract using signature-tagged mutagenesis. Microbiology.

[34] van Diemen P.M., Dziva F., Stevens M.P., Wallis T.S. (2005). Identification of enterohemorrhagic *Escherichia coli* O26:H- genes required for intestinal colonization in calves. Infect. Immun..

[35] Stevens M.P., Marches O., Campbell J., Huter V., Frankel G., Phillips A.D., Oswald E., Wallis T.S. (2002). Intimin, tir, and shiga toxin 1 do not influence enteropathogenic responses to shiga toxin-producing *Escherichia coli* in bovine ligated intestinal loops. Infect. Immun..

[36] Shaw D.J., Jenkins C., Pearce M.C., Cheasty T., Gunn G.J., Dougan G., Smith H.R., Woolhouse M.E., Frankel G. (2004). Shedding patterns of verocytotoxin-producing *Escherichia coli* strains in a cohort of calves and their dams on a Scottish beef farm. Appl. Environ. Microbiol..

[37] Wieler L.H., Bauerfeind R., Baljer G. (1992). Characterization of Shiga-like toxin producing *Escherichia coli* (SLTEC) isolated from calves with and without diarrhoea. Zentralbl. Bakteriol..

[38] Chanter N., Hall G.A., Bland A.P., Hayle A.J., Parsons K.R. (1986). Dysentery in calves caused by an atypical strain of *Escherichia coli* (S102-9). Vet. Microbiol..

[39] Dean-Nystrom E.A., Bosworth B.T., Cray W.C., Moon H.W. (1997). Pathogenicity of *Escherichia coli* O157:H7 in the intestines of neonatal calves. Infect. Immun..

[40] Janke B.H., Francis D.H., Collins J.E., Libal M.C., Zeman D.H., Johnson D.D., Neiger R.D. (1990). Attaching and effacing *Escherichia coli* infection as a cause of diarrhea in young calves. J. Am. Vet. Med. Assoc..

[41] Moxley R.A., Francis D.H. (1986). Natural and experimental infection with an attaching and effacing strain of *Escherichia coli* in calves. Infect. Immun..

[42] Richardson S.E., Karmali M.A., Becker L.E., Smith C.R. (1988). The histopathology of the hemolytic uremic syndrome associated with verocytotoxin-producing *Escherichia coli* infections. Hum. Pathol..

[43] van de Kar N.C., Monnens L.A., Karmali M.A., van Hinsbergh V.W. (1992). Tumor necrosis factor and interleukin-1 induce expression of the verocytotoxin receptor globotriaosylceramide on human endothelial cells: Implications for the pathogenesis of the hemolytic uremic syndrome. Blood.

[44] Proulx F., Seidman E.G., Karpman D. (2001). Pathogenesis of Shiga toxin-associated hemolytic uremic syndrome. Pediatr. Res..

[45] Pruimboom-Brees I.M., Morgan T.W., Ackermann M.R., Nystrom E.D., Samuel J.E., Cornick N.A., Moon H.W. (2000). Cattle lack vascular receptors for *Escherichia coli* O157:H7 Shiga toxins. Proc. Natl. Acad. Sci. USA.

[46] Mangeney M., Lingwood C.A., Taga S., Caillou B., Tursz T., Wiels J. (1993). Apoptosis induced in Burkitt’s lymphoma cells via Gb3/CD77, a glycolipid antigen. Cancer Res..

[47] Chark D., Nutikka A., Trusevych N., Kuzmina J., Lingwood C. (2004). Differential carbohydrate epitope recognition of globotriaosyl ceramide by verotoxins and a monoclonal antibody. Eur. J. Biochem..

[48] Schwarting G.A. (1980). Quantitative analysis of neutral glycosphingolipids from human lymphocyte subpopulations. Biochem. J..

[49] Taga S., Tetaud C., Mangeney M., Tursz T., Wiels J. (1995). Sequential changes in glycolipid expression during human B cell differentiation: Enzymatic bases. Biochim. Biophys. Acta.

[50] Menge C., Stamm I., Wuhrer M., Geyer R., Wieler L.H., Baljer G. (2001). Globotriaosylceramide (Gb(3)/CD77) is synthesized and surface expressed by bovine lymphocytes upon activation in vitro. Vet. Immunol. Immunopathol..

[51] Menge C., Wieler L.H., Schlapp T., Baljer G. (1999). Shiga toxin 1 from *Escherichia coli* blocks activation and proliferation of bovine lymphocyte subpopulations in vitro. Infect. Immun..

[52] Chu A. (2010). Immunomodulation by Shiga Toxin 2. Ph.D. Thesis.

[53] Mangeney M., Richard Y., Coulaud D., Tursz T., Wiels J. (1991). CD77: An antigen of germinal center B cells entering apoptosis. Eur. J. Immunol.

[54] Mangeney M., Rousselet G., Taga S., Tursz T., Wiels J. (1995). The fate of human CD77+ germinal center B lymphocytes after rescue from apoptosis. Mol. Immunol..

[55] Menge C., Stamm I., Blessenohl M., Wieler L.H., Baljer G. (2003). Verotoxin 1 from *Escherichia coli* affects Gb3/CD77+ bovine lymphocytes independent of interleukin-2, tumor necrosis factor-alpha, and interferon-alpha. Exp. Biol. Med. (Maywood).

[56] Menge C., Blessenohl M., Eisenberg T., Stamm I., Baljer G. (2004). Bovine ileal intraepithelial lymphocytes represent target cells for Shiga toxin 1 from *Escherichia coli*. Infect. Immun..

[57] Wiels J., Fellous M., Tursz T. (1981). Monoclonal antibody against a Burkitt lymphoma-associated antigen. Proc. Natl. Acad. Sci. USA.

[58] Liu Y.J., Barthelemy C., de Bouteiller O., Banchereau J. (1994). The differences in survival and phenotype between centroblasts and centrocytes. Adv. Exp. Med. Biol..

[59] Cohen A., Madrid-Marina V., Estrov Z., Freedman M.H., Lingwood C.A., Dosch H.M. (1990). Expression of glycolipid receptors to Shiga-like toxin on human B lymphocytes: A mechanism for the failure of long-lived antibody response to dysenteric disease. Int. Immunol..

[60] Ramegowda B., Tesh V.L. (1996). Differentiation-associated toxin receptor modulation, cytokine production, and sensitivity to Shiga-like toxins in human monocytes and monocytic cell lines. Infect. Immun..

[61] Kniep B., Monner D.A., Schwulera U., Muhlradt P.F. (1985). Glycosphingolipids of the globo-series are associated with the monocytic lineage of human myeloid cells. Eur. J. Biochem..

[62] Pudymaitis A., Lingwood C.A. (1992). Susceptibility to verotoxin as a function of the cell cycle. J. Cell Physiol..

[63] Stamm I., Wuhrer M., Geyer R., Baljer G., Menge C. (2002). Bovine lymphocytes express functional receptors for *Escherichia coli* Shiga toxin 1. Microb. Pathog..

[64] Lindberg A.A., Brown J.E., Stromberg N., Westling-Ryd M., Schultz J.E., Karlsson K.A. (1987). Identification of the carbohydrate receptor for Shiga toxin produced by Shigella dysenteriae type 1. J. Biol. Chem..

[65] Pellizzari A., Pang H., Lingwood C.A. (1992). Binding of verocytotoxin 1 to its receptor is influenced by differences in receptor fatty acid content. Biochemistry.

[66] Christopher-Hennings J., Willgohs J.A., Francis D.H., Raman U.A., Moxley R.A., Hurley D.J. (1993). Immunocompromise in gnotobiotic pigs induced by verotoxin-producing *Escherichia coli* (O111:NM). Infect. Immun..

[67] Sugatani J., Igarashi T., Shimura M., Yamanaka T., Takeda T., Miwa M. (2000). Disorders in the immune responses of T- and B-cells in mice administered intravenous verotoxin 2. Life Sci..

[68] Eiklid K., Olsnes S. (1980). Interaction of Shigella shigae cytotoxin with receptors on sensitive and insensitive cells. J. Recept. Res..

[69] Ghislain J., Lingwood C.A., Fish E.N. (1994). Evidence for glycosphingolipid modification of the type 1 IFN receptor. J. Immunol..

[70] Klapproth J.M., Donnenberg M.S., Abraham J.M., Mobley H.L., James S.P. (1995). Products of enteropathogenic *Escherichia coli* inhibit lymphocyte activation and lymphokine production. Infect. Immun..

[71] Klapproth J.M., Scaletsky I.C., McNamara B.P., Lai L.C., Malstrom C., James S.P., Donnenberg M.S. (2000). A large toxin from pathogenic *Escherichia coli* strains that inhibits lymphocyte activation. Infect. Immun..

[72] Kerner K., Bridger P.S., Kopf G., Frohlich J., Barth S., Willems H., Bauerfeind R., Baljer G., Menge C. (2015). Evaluation of biological safety in vitro and immunogenicity in vivo of recombinant *Escherichia coli* Shiga toxoids as candidate vaccines in cattle. Vet. Res..

[73] Obrig T.G., Moran T.P., Brown J.E. (1987). The mode of action of Shiga toxin on peptide elongation of eukaryotic protein synthesis. Biochem. J..

[74] Harrison L.M., van Haaften W.C., Tesh V.L. (2004). Regulation of proinflammatory cytokine expression by Shiga toxin 1 and/or lipopolysaccharides in the human monocytic cell line THP-1. Infect. Immun..

[75] Taga S., Carlier K., Mishal Z., Capoulade C., Mangeney M., Lecluse Y., Coulaud D., Tetaud C., Pritchard L.L., Tursz T. (1997). Intracellular signaling events in CD77-mediated apoptosis of Burkitt’s lymphoma cells. Blood.

[76] Katagiri Y.U., Kiyokawa N., Fujimoto J. (2001). The effect of shiga toxin binding to globotriaosylceramidein rafts of human kidney cells and Burkitt’slymphoma cells. Trends Glycosci. Glycotech..

[77] Matthews K.R., Murdough P.A., Bramley A.J. (1997). Invasion of bovine epithelial cells by verocytotoxin-producing *Escherichia coli* O157:H7. J. Appl. Microbiol..

[78] Schmidt N., Barth S.A., Frahm J., Meyer U., Danicke S., Geue L., Menge C. (2018). Decreased STEC shedding by cattle following passive and active vaccination based on recombinant *Escherichia coli* Shiga toxoids. Vet. Res..

[79] Waters W.R., Harp J.A., Nonnecke B.J. (1995). Phenotypic analysis of peripheral blood lymphocytes and intestinal intra-epithelial lymphocytes in calves. Vet. Immunol. Immunopathol..

[80] Fitzgerald S.F., Beckett A.E., Palarea-Albaladejo J., McAteer S., Shaaban S., Morgan J., Ahmad N.I., Young R., Mabbott N.A., Morrison L. (2019). Shiga toxin sub-type 2a increases the efficiency of *Escherichia coli* O157 transmission between animals and restricts epithelial regeneration in bovine enteroids. PLoS Pathog..

[81] Hoey D.E., Currie C., Else R.W., Nutikka A., Lingwood C.A., Gally D.L., Smith D.G. (2002). Expression of receptors for verotoxin 1 from *Escherichia coli* O157 on bovine intestinal epithelium. J. Med. Microbiol..

[82] Moussay E., Stamm I., Taubert A., Baljer G., Menge C. (2006). *Escherichia coli* Shiga toxin 1 enhances il-4 transcripts in bovine ileal intraepithelial lymphocytes. Vet. Immunol. Immunopathol..

[83] Grogan J.L., Mohrs M., Harmon B., Lacy D.A., Sedat J.W., Locksley R.M. (2001). Early transcription and silencing of cytokine genes underlie polarization of T helper cell subsets. Immunity.

[84] Winzen R., Kracht M., Ritter B., Wilhelm A., Chen C.Y., Shyu A.B., Muller M., Gaestel M., Resch K., Holtmann H. (1999). The p38 MAP kinase pathway signals for cytokine-induced mRNA stabilization via MAP kinase-activated protein kinase 2 and an AU-rich region-targeted mechanism. EMBO J..

[85] Thorpe C.M., Hurley B.P., Lincicome L.L., Jacewicz M.S., Keusch G.T., Acheson D.W. (1999). Shiga toxins stimulate secretion of interleukin-8 from intestinal epithelial cells. Infect. Immun..

[86] Thorpe C.M., Smith W.E., Hurley B.P., Acheson D.W. (2001). Shiga toxins induce, superinduce, and stabilize a variety of C-X-C chemokine mRNAs in intestinal epithelial cells, resulting in increased chemokine expression. Infect. Immun..

[87] Jacobson A., Peltz S.W. (1996). Interrelationships of the pathways of mRNA decay and translation in eukaryotic cells. Annu. Rev. Biochem..

[88] Rook G.A., Hernandez-Pando R., Dheda K., Teng Seah G. (2004). IL-4 in tuberculosis: Implications for vaccine design. Trends Immunol..

[89] Bitzan M.M., Wang Y., Lin J., Marsden P.A. (1998). Verotoxin and ricin have novel effects on preproendothelin-1 expression but fail to modify nitric oxide synthase (ecNOS) expression and NO production in vascular endothelium. J. Clin. Investig..

[90] Stein G.M., Pfuller U., Schietzel M., Bussing A. (2000). Expression of interleukin-4 in apoptotic cells: Stimulation of the type-2 cytokine by different toxins in human peripheral blood mononuclear and tumor cells. Cytometry.

[91] Ebert E.C., Roberts A.I. (1996). IL-4 down-regulates the responsiveness of human intraepithelial lymphocytes. Clin. Exp. Immunol..

[92] Menge C., Stamm I., Van Diemen P.M., Sopp P., Baljer G., Wallis T.S., Stevens M.P. (2004). Phenotypic and functional characterization of intraepithelial lymphocytes in a bovine ligated intestinal loop model of enterohaemorrhagic *Escherichia coli* infection. J. Med. Microbiol..

[93] Menge C., Loos D., Bridger P.S., Barth S., Werling D., Baljer G. (2015). Bovine macrophages sense *Escherichia coli* Shiga toxin 1. Innate Immun..

[94] Cameron P., Paton N., Smith D.G. (2012). Verotoxin-2 activates mitogen-activated protein kinases in bovine adherent peripheral blood mononuclear cells. J. Comp. Pathol..

[95] Cameron P., Smith S.J., Giembycz M.A., Rotondo D., Plevin R. (2003). Verotoxin activates mitogen-activated protein kinase in human peripheral blood monocytes: Role in apoptosis and proinflammatory cytokine release. Br. J. Pharmacol..

[96] Olsnes S., Reisbig R., Eiklid K. (1981). Subunit structure of Shigella cytotoxin. J. Biol. Chem..

[97] Mosser D.M. (2003). The many faces of macrophage activation. J. Leukoc. Biol..

[98] Mosser D.M., Edwards J.P. (2008). Exploring the full spectrum of macrophage activation. Nat. Rev. Immunol..

[99] Stamm I., Mohr M., Bridger P.S., Schropfer E., Konig M., Stoffregen W.C., Dean-Nystrom E.A., Baljer G., Menge C. (2008). Epithelial and mesenchymal cells in the bovine colonic mucosa differ in their responsiveness to *Escherichia coli* Shiga toxin 1. Infect. Immun..

[100] Liu J., Akahoshi T., Sasahana T., Kitasato H., Namai R., Sasaki T., Inoue M., Kondo H. (1999). Inhibition of neutrophil apoptosis by verotoxin 2 derived from *Escherichia coli* O157:H7. Infect. Immun..

[101] King A.J., Sundaram S., Cendoroglo M., Acheson D.W., Keusch G.T. (1999). Shiga toxin induces superoxide production in polymorphonuclear cells with subsequent impairment of phagocytosis and responsiveness to phorbol esters. J. Infect. Dis..

[102] Yamasaki C., Natori Y., Zeng X.T., Ohmura M., Yamasaki S., Takeda Y. (1999). Induction of cytokines in a human colon epithelial cell line by Shiga toxin 1 (Stx1) and Stx2 but not by non-toxic mutant Stx1 which lacks N-glycosidase activity. FEBS Lett..

[103] Hurley B.P., Thorpe C.M., Acheson D.W. (2001). Shiga toxin translocation across intestinal epithelial cells is enhanced by neutrophil transmigration. Infect. Immun..

[104] te Loo D.M., Monnens L.A., van Der Velden T.J., Vermeer M.A., Preyers F., Demacker P.N., van Den Heuvel L.P., van Hinsbergh V.W. (2000). Binding and transfer of verocytotoxin by polymorphonuclear leukocytes in hemolytic uremic syndrome. Blood.

[105] Svanborg C., Godaly G., Hedlund M. (1999). Cytokine responses during mucosal infections: Role in disease pathogenesis and host defence. Curr. Opin. Microbiol..

[106] Schoonderwoerd M., Clarke R.C., van Dreumel A.A., Rawluk S.A. (1988). Colitis in calves: Natural and experimental infection with a verotoxin-producing strain of *Escherichia coli* O111:NM. Can. J. Vet. Res..

[107] Diez-Fraile A., Meyer E., Massart-Leen A.M., Burvenich C. (2000). Effect of isoproterenol and dexamethasone on the lipopolysaccharide induced expression of CD11b on bovine neutrophils. Vet. Immunol. Immunopathol..

[108] Menge C., Eisenberg T., Stamm I., Baljer G. (2006). Comparison of binding and effects of *Escherichia coli* Shiga toxin 1 on bovine and ovine granulocytes. Vet. Immunol. Immunopathol..

[109] Mobassaleh M., Donohue-Rolfe A., Jacewicz M., Grand R.J., Keusch G.T. (1988). Pathogenesis of shigella diarrhea: Evidence for a developmentally regulated glycolipid receptor for shigella toxin involved in the fluid secretory response of rabbit small intestine. J. Infect. Dis..

[110] Smits E., Burvenich C., Guidry A.J., Heyneman R., Massart-Leen A. (1999). Diapedesis across mammary epithelium reduces phagocytic and oxidative burst of bovine neutrophils. Vet. Immunol. Immunopathol..

[111] Mohr M. (2007). Nachweis und Reaktivität epithelialer und mesenchymaler Zielzellen für Escherichia coli Shigatoxin in den Kolonkrypten des Rindes.

[112] Hoey D.E., Sharp L., Currie C., Lingwood C.A., Gally D.L., Smith D.G. (2003). Verotoxin 1 binding to intestinal crypt epithelial cells results in localization to lysosomes and abrogation of toxicity. Cell. Microbiol..

[113] Schuller S., Frankel G., Phillips A.D. (2004). Interaction of Shiga toxin from *Escherichia coli* with human intestinal epithelial cell lines and explants: Stx2 induces epithelial damage in organ culture. Cell. Microbiol..

[114] Lingwood C.A. (1999). Glycolipid receptors for verotoxin and Helicobacter pylori: Role in pathology. Biochim. Biophys. Acta.

[115] Falguieres T., Mallard F., Baron C., Hanau D., Lingwood C., Goud B., Salamero J., Johannes L. (2001). Targeting of Shiga toxin B-subunit to retrograde transport route in association with detergent-resistant membranes. Mol. Biol. Cell.

[116] Tesh V.L., Ramegowda B., Samuel J.E. (1994). Purified Shiga-like toxins induce expression of proinflammatory cytokines from murine peritoneal macrophages. Infect. Immun..

[117] van Setten P.A., Monnens L.A., Verstraten R.G., van den Heuvel L.P., van Hinsbergh V.W. (1996). Effects of verocytotoxin-1 on nonadherent human monocytes: Binding characteristics, protein synthesis, and induction of cytokine release. Blood.

[118] Vlisidou I., Lyte M., van Diemen P.M., Hawes P., Monaghan P., Wallis T.S., Stevens M.P. (2004). The neuroendocrine stress hormone norepinephrine augments *Escherichia coli* O157:H7-induced enteritis and adherence in a bovine ligated ileal loop model of infection. Infect. Immun..

[119] Stevens M.P., Roe A.J., Vlisidou I., van Diemen P.M., La Ragione R.M., Best A., Woodward M.J., Gally D.L., Wallis T.S. (2004). Mutation of toxB and a truncated version of the efa-1 gene in *Escherichia coli* O157:H7 influences the expression and secretion of locus of enterocyte effacement-encoded proteins but not intestinal colonization in calves or sheep. Infect. Immun..

[120] Stevens M.P., van Diemen P.M., Frankel G., Phillips A.D., Wallis T.S. (2002). Efa1 influences colonization of the bovine intestine by shiga toxin-producing *Escherichia coli* serotypes O5 and O111. Infect. Immun..

[121] Bridger P.S., Mohr M., Stamm I., Frohlich J., Follmann W., Birkner S., Metcalfe H., Werling D., Baljer G., Menge C. (2010). Primary bovine colonic cells: A model to study strain-specific responses to *Escherichia coli*. Vet. Immunol. Immunopathol..

[122] Hauf N., Chakraborty T. (2003). Suppression of NF-kappa B activation and proinflammatory cytokine expression by Shiga toxin-producing *Escherichia coli*. J. Immunol..

[123] Stalb S., Barth S.A., Sobotta K., Liebler-Tenorio E., Geue L., Menge C. (2018). Pro-inflammatory capacity of *Escherichia coli* O104:H4 outbreak strain during colonization of intestinal epithelial cells from human and cattle. Int. J. Med. Microbiol..

[124] Barth S.A., Menge C., Eichhorn I., Semmler T., Wieler L.H., Pickard D., Belka A., Berens C., Geue L. (2016). The Accessory Genome of Shiga Toxin-Producing *Escherichia coli* Defines a Persistent Colonization Type in Cattle. Appl. Environ. Microbiol..

[125] Pradhan S., Karve S.S., Weiss A.A., Hawkins J., Poling H.M., Helmrath M.A., Wells J.M., McCauley H.A. (2020). Tissue Responses to Shiga Toxin in Human Intestinal Organoids. Cell Mol. Gastroenterol. Hepatol..

[126] Ferens W.A., Hovde C.J. (2000). Antiviral activity of shiga toxin 1: Suppression of bovine leukemia virus-related spontaneous lymphocyte proliferation. Infect. Immun..

[127] Barrett T.A., Gajewski T.F., Danielpour D., Chang E.B., Beagley K.W., Bluestone J.A. (1992). Differential function of intestinal intraepithelial lymphocyte subsets. J. Immunol..

[128] Suzuki R., Nakao A., Kanamaru Y., Okumura K., Ogawa H., Ra C. (2002). Localization of intestinal intraepithelial T lymphocytes involves regulation of alphaEbeta7 expression by transforming growth factor-beta. Int. Immunol..

[129] Corbishley A., Ahmad N.I., Hughes K., Hutchings M.R., McAteer S.P., Connelley T.K., Brown H., Gally D.L., McNeilly T.N. (2014). Strain-dependent cellular immune responses in cattle following *Escherichia coli* O157:H7 colonization. Infect. Immun..

[130] Xue Y., Zhang H., Wang H., Hu J., Du M., Zhu M.J. (2014). Host inflammatory response inhibits *Escherichia coli* O157:H7 adhesion to gut epithelium through augmentation of mucin expression. Infect. Immun..

[131] Nart P., Naylor S.W., Huntley J.F., McKendrick I.J., Gally D.L., Low J.C. (2008). Responses of cattle to gastrointestinal colonization by *Escherichia coli* O157:H7. Infect. Immun..

[132] Kieckens E., Rybarczyk J., Li R.W., Vanrompay D., Cox E. (2016). Potential immunosuppressive effects of *Escherichia coli* O157:H7 experimental infection on the bovine host. BMC Genom..

[133] Wang O., McAllister T.A., Plastow G., Stanford K., Selinger B., Guan L.L. (2017). Host mechanisms involved in cattle *Escherichia coli* O157 shedding: A fundamental understanding for reducing foodborne pathogen in food animal production. Sci. Rep..

[134] Wang O., Liang G., McAllister T.A., Plastow G., Stanford K., Selinger B., Guan le L. (2016). Comparative Transcriptomic Analysis of Rectal Tissue from Beef Steers Revealed Reduced Host Immunity in *Escherichia coli* O157:H7 Super-Shedders. PLoS ONE.

[135] Wieler L.H., Franke S., Menge C., Rose M., Bauerfeind R., Karch H., Baljer G. (1995). The immune response in edema disease of weaned piglets measured with a recombinant B subunit of shiga-like toxin II. Dtsch. Tierarztl. Wochenschr..

[136] Reymond D., Johnson R.P., Karmali M.A., Petric M., Winkler M., Johnson S., Rahn K., Renwick S., Wilson J., Clarke R.C. (1996). Neutralizing antibodies to *Escherichia coli* Vero cytotoxin 1 and antibodies to O157 lipopolysaccharide in healthy farm family members and urban residents. J. Clin. Microbiol..

[137] Bretschneider G., Berberov E.M., Moxley R.A. (2007). Isotype-specific antibody responses against *Escherichia coli* O157:H7 locus of enterocyte effacement proteins in adult beef cattle following experimental infection. Vet. Immunol. Immunopathol..

[138] Johnson R.P., Cray W.C., Johnson S.T. (1996). Serum antibody responses of cattle following experimental infection with *Escherichia coli* O157:H7. Infect. Immun..

[139] Frohlich J., Baljer G., Menge C. (2009). Maternally and naturally acquired antibodies to Shiga toxins in a cohort of calves shedding Shiga-toxigenic *Escherichia coli*. Appl. Environ. Microbiol..

[140] Kuribayashi T., Seita T., Matsumoto M., Furuhata K., Tagata K., Yamamoto S. (2009). Bovine colostral antibody against verotoxin 2 derived from *Escherichia coli* O157:H7: Resistance to proteases and effects in beagle dogs. Comp. Med..

[141] Tetsurou S., Kuribayashi T., Fukuyama M., Yamaguchi S., Yamamoto S. (2015). Inhibition of verotoxin (VT) 2 absorption into systemic blood from intestine by repeated administration of bovine immune colostral antibody against VT2 in mice. J. Microbiol. Immunol. Infect..

[142] Albanese A., Sacerdoti F., Seyahian E.A., Amaral M.M., Fiorentino G., Fernandez Brando R., Vilte D.A., Mercado E.C., Palermo M.S., Cataldi A. (2018). Immunization of pregnant cows with Shiga toxin-2 induces high levels of specific colostral antibodies and lactoferrin able to neutralize E. coli O157:H7 pathogenicity. Vaccine.

[143] Corbishley A., Connelley T.K., Wolfson E.B., Ballingall K., Beckett A.E., Gally D.L., McNeilly T.N. (2016). Identification of epitopes recognised by mucosal CD4(+) T-cell populations from cattle experimentally colonised with *Escherichia coli* O157:H7. Vet. Res..

[144] Bretschneider G., Berberov E.M., Moxley R.A. (2008). Enteric mucosal antibodies to *Escherichia coli* O157:H7 in adult cattle. Vet. Rec..

[145] Beckett A.E. (2018). Defining the impact of colonisation with Shiga toxin positive E. coli O157 on adaptive immunity in cattle. Ph.D. Thesis.

[146] Smith D.G., Naylor S.W., Gally D.L. (2002). Consequences of EHEC colonisation in humans and cattle. Int. J. Med. Microbiol..

[147] Fiocchi C. (1997). Intestinal inflammation: A complex interplay of immune and nonimmune cell interactions. Am. J. Physiol..

[148] Wieler L.H., Schwanitz A., Vieler E., Busse B., Steinruck H., Kaper J.B., Baljer G. (1998). Virulence properties of Shiga toxin-producing *Escherichia coli* (STEC) strains of serogroup O118, a major group of STEC pathogens in calves. J. Clin. Microbiol..

[149] Simmons C.P., Clare S., Dougan G. (2001). Understanding mucosal responsiveness: Lessons from enteric bacterial pathogens. Semin. Immunol..

[150] Borman-Eby H.C., McEwen S.A., Clarke R.C., McNab W.B., Rahn K., Valdivieso-Garcia A. (1993). The seroprevalence of verocytotoxin-producing *Escherichia coli* in Ontario dairy cows and associations with production and management. Prev. Vet. Med..

[151] Eisenberg T. (2003). Untersuchungen zur Wirkung von Shigatoxin 1 von Escherichia coli auf Zellen der unspezifischen Immunabwehr bei Rind, Schaf und Ziege.

[152] Pohlenz J.F., Winter K.R., Dean-Nystrom E.A. (2005). Shiga-toxigenic *Escherichia coli*-inoculated neonatal piglets develop kidney lesions that are comparable to those in humans with hemolytic-uremic syndrome. Infect. Immun..

[153] Winter K.R., Stoffregen W.C., Dean-Nystrom E.A. (2004). Shiga toxin binding to isolated porcine tissues and peripheral blood leukocytes. Infect. Immun..

[154] Inobe J., Slavin A.J., Komagata Y., Chen Y., Liu L., Weiner H.L. (1998). IL-4 is a differentiation factor for transforming growth factor-beta secreting Th3 cells and oral administration of IL-4 enhances oral tolerance in experimental allergic encephalomyelitis. Eur. J. Immunol..

[155] Mennechet F.J., Kasper L.H., Rachinel N., Minns L.A., Luangsay S., Vandewalle A., Buzoni-Gatel D. (2004). Intestinal intraepithelial lymphocytes prevent pathogen-driven inflammation and regulate the Smad/T-bet pathway of lamina propria CD4+ T cells. Eur. J. Immunol..

[156] Naylor S.W., Roe A.J., Nart P., Spears K., Smith D.G., Low J.C., Gally D.L. (2005). *Escherichia coli* O157: H7 forms attaching and effacing lesions at the terminal rectum of cattle and colonization requires the LEE4 operon. Microbiology.

[157] Hamm K., Barth S.A., Stalb S., Geue L., Liebler-Tenorio E., Teifke J.P., Lange E., Tauscher K., Kotterba G., Bielaszewska M. (2016). Experimental Infection of Calves with *Escherichia coli* O104:H4 outbreak strain. Sci. Rep..

[158] Stevens M.P., van Diemen P.M., Dziva F., Jones P.W., Wallis T.S. (2002). Options for the control of enterohaemorrhagic *Escherichia coli* in ruminants. Microbiology.

[159] Asper D.J., Karmali M.A., Townsend H., Rogan D., Potter A.A. (2011). Serological response of Shiga toxin-producing *Escherichia coli* type III secreted proteins in sera from vaccinated rabbits, naturally infected cattle, and humans. Clin. Vaccine Immunol..

[160] Potter A.A., Klashinsky S., Li Y., Frey E., Townsend H., Rogan D., Erickson G., Hinkley S., Klopfenstein T., Moxley R.A. (2004). Decreased shedding of *Escherichia coli* O157:H7 by cattle following vaccination with type III secreted proteins. Vaccine.

[161] Allen K.J., Rogan D., Finlay B.B., Potter A.A., Asper D.J. (2011). Vaccination with type III secreted proteins leads to decreased shedding in calves after experimental infection with *Escherichia coli* O157. Can. J. Vet. Res..

[162] Sheng H., Lim J.Y., Knecht H.J., Li J., Hovde C.J. (2006). Role of *Escherichia coli* O157:H7 virulence factors in colonization at the bovine terminal rectal mucosa. Infect. Immun..

[163] Cornick N.A., Booher S.L., Casey T.A., Moon H.W. (2000). Persistent colonization of sheep by *Escherichia coli* O157:H7 and other E. coli pathotypes. Appl. Environ. Microbiol..

[164] Cornick N.A., Helgerson A.F., Sharma V. (2007). Shiga toxin and Shiga toxin-encoding phage do not facilitate *Escherichia coli* O157:H7 colonization in sheep. Appl. Environ. Microbiol..

[165] Wang L.Y.R., Jokinen C.C., Laing C.R., Johnson R.P., Ziebell K., Gannon V.P.J. (2020). Assessing the genomic relatedness and evolutionary rates of persistent verotoxigenic *Escherichia coli* serotypes within a closed beef herd in Canada. Microb. Genom..

[166] Cabal A., Geue L., Gomez-Barrero S., Barth S., Barcena C., Hamm K., Porrero M.C., Valverde A., Canton R., Menge C. (2015). Detection of virulence-associated genes characteristic of intestinal *Escherichia coli* pathotypes, including the enterohemorrhagic/enteroaggregative O104:H4, in bovines from Germany and Spain. Microbiol. Immunol..

[167] Bai J., McAteer S.P., Paxton E., Mahajan A., Gally D.L., Tree J.J. (2011). Screening of an E. coli O157:H7 Bacterial Artificial Chromosome Library by Comparative Genomic Hybridization to Identify Genomic Regions Contributing to Growth in Bovine Gastrointestinal Mucus and Epithelial Cell Colonization. Front. Microbiol..

[168] Ogura Y., Ooka T., Iguchi A., Toh H., Asadulghani M., Oshima K., Kodama T., Abe H., Nakayama K., Kurokawa K. (2009). Comparative genomics reveal the mechanism of the parallel evolution of O157 and non-O157 enterohemorrhagic *Escherichia coli*. Proc. Natl. Acad. Sci. USA.

[169] Hayashi T., Makino K., Ohnishi M., Kurokawa K., Ishii K., Yokoyama K., Han C.G., Ohtsubo E., Nakayama K., Murata T. (2001). Complete genome sequence of enterohemorrhagic *Escherichia coli* O157:H7 and genomic comparison with a laboratory strain K-12. DNA Res..

[170] Perna N.T., Plunkett G., Burland V., Mau B., Glasner J.D., Rose D.J., Mayhew G.F., Evans P.S., Gregor J., Kirkpatrick H.A. (2001). Genome sequence of enterohaemorrhagic *Escherichia coli* O157:H7. Nature.

[171] Martorelli L., Hovde C.J., Vilte D.A., Albanese A., Zotta E., Ibarra C., Cantet R.J., Mercado E.C., Cataldi A. (2015). Impact of Infection Dose and Previous Serum Antibodies against the Locus of Enterocyte Effacement Proteins on *Escherichia coli* O157:H7 Shedding in Calves following Experimental Infection. Biomed. Res. Int..

[172] Vilte D.A., Larzabal M., Garbaccio S., Gammella M., Rabinovitz B.C., Elizondo A.M., Cantet R.J., Delgado F., Meikle V., Cataldi A. (2011). Reduced faecal shedding of *Escherichia coli* O157:H7 in cattle following systemic vaccination with gamma-intimin C(2)(8)(0) and EspB proteins. Vaccine.

[173] McNeilly T.N., Mitchell M.C., Corbishley A., Nath M., Simmonds H., McAteer S.P., Mahajan A., Low J.C., Smith D.G., Huntley J.F. (2015). Optimizing the Protection of Cattle against *Escherichia coli* O157:H7 Colonization through Immunization with Different Combinations of H7 Flagellin, Tir, Intimin-531 or EspA. PLoS ONE.

[174] Klapproth J.M., Donnenberg M.S., Abraham J.M., James S.P. (1996). Products of enteropathogenic E. coli inhibit lymphokine production by gastrointestinal lymphocytes. Am. J. Physiol..

[175] Malstrom C., James S. (1998). Inhibition of murine splenic and mucosal lymphocyte function by enteric bacterial products. Infect. Immun..

[176] Deacon V., Dziva F., van Diemen P.M., Frankel G., Stevens M.P. (2010). Efa-1/LifA mediates intestinal colonization of calves by enterohaemorrhagic *Escherichia coli* O26: H- in a manner independent of glycosyltransferase and cysteine protease motifs or effects on type III secretion. Microbiology.

[177] Cassady-Cain R.L., Hope J.C., Stevens M.P. (2018). Direct Manipulation of T Lymphocytes by Proteins of Gastrointestinal Bacterial Pathogens. Infect. Immun..

[178] Cassady-Cain R.L., Blackburn E.A., Bell C.R., Elshina E., Hope J.C., Stevens M.P. (2017). Inhibition of Antigen-Specific and Nonspecific Stimulation of Bovine T and B Cells by Lymphostatin from Attaching and Effacing *Escherichia coli*. Infect. Immun..

[179] Cassady-Cain R.L., Blackburn E.A., Alsarraf H., Dedic E., Bease A.G., Bottcher B., Jorgensen R., Wear M., Stevens M.P. (2016). Biophysical Characterization and Activity of Lymphostatin, a Multifunctional Virulence Factor of Attaching and Effacing *Escherichia coli*. J. Biol. Chem..

[180] Higgins L.M., Frankel G., Douce G., Dougan G., MacDonald T.T. (1999). Citrobacter rodentium infection in mice elicits a mucosal Th1 cytokine response and lesions similar to those in murine inflammatory bowel disease. Infect. Immun..

[181] Bohle S.M. (2006). Investigations concerning the immunomodulatory effect of the virulence factor “intimin” of enteropathogenic and enterohemorrhagic Escherichia coli in cattle.

[182] Rashid R.A., Tabata T.A., Oatley M.J., Besser T.E., Tarr P.I., Moseley S.L. (2006). Expression of putative virulence factors of *Escherichia coli* O157:H7 differs in bovine and human infections. Infect. Immun..

[183] Ritchie J.M., Wagner P.L., Acheson D.W., Waldor M.K. (2003). Comparison of Shiga toxin production by hemolytic-uremic syndrome-associated and bovine-associated Shiga toxin-producing *Escherichia coli* isolates. Appl. Environ. Microbiol..

[184] Lejeune J.T., Abedon S.T., Takemura K., Christie N.P., Sreevatsan S. (2004). Human *Escherichia coli* O157:H7 genetic marker in isolates of bovine origin. Emerg Infect. Dis..

[185] Lupolova N., Dallman T.J., Matthews L., Bono J.L., Gally D.L. (2016). Support vector machine applied to predict the zoonotic potential of E. coli O157 cattle isolates. Proc. Natl. Acad. Sci. USA.

[186] Chase-Topping M., Gally D., Low C., Matthews L., Woolhouse M. (2008). Super-shedding and the link between human infection and livestock carriage of *Escherichia coli* O157. Nat Rev Microbiol.

[187] Xu X., McAteer S.P., Tree J.J., Shaw D.J., Wolfson E.B., Beatson S.A., Roe A.J., Allison L.J., Chase-Topping M.E., Mahajan A. (2012). Lysogeny with Shiga toxin 2-encoding bacteriophages represses type III secretion in enterohemorrhagic *Escherichia coli*. PLoS Pathog..

[188] Furniss R.C.D., Clements A. (2018). Regulation of the Locus of Enterocyte Effacement in Attaching and Effacing Pathogens. J. Bacteriol..

[189] Hughes D.T., Clarke M.B., Yamamoto K., Rasko D.A., Sperandio V. (2009). The QseC adrenergic signaling cascade in Enterohemorrhagic E. coli (EHEC). PLoS Pathog..

[190] Parker C.T., Russell R., Njoroge J.W., Jimenez A.G., Taussig R., Sperandio V. (2017). Genetic and Mechanistic Analyses of the Periplasmic Domain of the Enterohemorrhagic *Escherichia coli* QseC Histidine Sensor Kinase. J. Bacteriol..

[191] Garmendia J., Phillips A.D., Carlier M.F., Chong Y., Schuller S., Marches O., Dahan S., Oswald E., Shaw R.K., Knutton S. (2004). TccP is an enterohaemorrhagic *Escherichia coli* O157:H7 type III effector protein that couples Tir to the actin-cytoskeleton. Cell. Microbiol..

[192] Pacheco A.R., Curtis M.M., Ritchie J.M., Munera D., Waldor M.K., Moreira C.G., Sperandio V. (2012). Fucose sensing regulates bacterial intestinal colonization. Nature.

[193] Berger P., Kouzel I.U., Berger M., Haarmann N., Dobrindt U., Koudelka G.B., Mellmann A. (2019). Carriage of Shiga toxin phage profoundly affects *Escherichia coli* gene expression and carbon source utilization. BMC Genom..

[194] Su L.K., Lu C.P., Wang Y., Cao D.M., Sun J.H., Yan Y.X. (2010). Lysogenic infection of a Shiga toxin 2-converting bacteriophage changes host gene expression, enhances host acid resistance and motility. Mol. Biol. (Mosk).

[195] Veses-Garcia M., Liu X., Rigden D.J., Kenny J.G., McCarthy A.J., Allison H.E. (2015). Transcriptomic analysis of Shiga-toxigenic bacteriophage carriage reveals a profound regulatory effect on acid resistance in *Escherichia coli*. Appl. Environ. Microbiol..

[196] Hernandez-Doria J.D., Sperandio V. (2018). Bacteriophage Transcription Factor Cro Regulates Virulence Gene Expression in Enterohemorrhagic *Escherichia coli*. Cell Host Microbe.

[197] Barth S.A., Weber M., Schaufler K., Berens C., Geue L., Menge C. (2020). Metabolic Traits of Bovine Shiga Toxin-Producing *Escherichia coli* (STEC) Strains with Different Colonization Properties. Toxins.

[198] Reeves P. (1995). Role of O-antigen variation in the immune response. Trends Microbiol..

[199] Bazaka K., Crawford R.J., Nazarenko E.L., Ivanova E.P. (2011). Bacterial extracellular polysaccharides. Adv. Exp. Med. Biol..

[200] Wang L., Qu W., Reeves P.R. (2001). Sequence analysis of four Shigella boydii O-antigen loci: Implication for *Escherichia coli* and Shigella relationships. Infect. Immun..

[201] Geue L., Menge C., Eichhorn I., Semmler T., Wieler L.H., Pickard D., Berens C., Barth S.A. (2017). Evidence for Contemporary Switching of the O-Antigen Gene Cluster between Shiga Toxin-Producing *Escherichia coli* Strains Colonizing Cattle. Front. Microbiol..

[202] Basu I., Ferens W.A., Stone D.M., Hovde C.J. (2003). Antiviral activity of shiga toxin requires enzymatic activity and is associated with increased permeability of the target cells. Infect. Immun..

[203] Ferens W.A., Hovde C.J. (2007). The non-toxic A subunit of Shiga toxin type 1 prevents replication of bovine immunodeficiency virus in infected cells. Virus Res..

[204] Ferens W.A., Halver M., Gustin K.E., Ott T., Hovde C.J. (2007). Differential sensitivity of viruses to the antiviral activity of Shiga toxin 1 A subunit. Virus Res..

[205] Ferens W.A., Cobbold R., Hovde C.J. (2006). Intestinal Shiga toxin-producing *Escherichia coli* bacteria mitigate bovine leukemia virus infection in experimentally infected sheep. Infect. Immun..

[206] Ferens W.A., Haruna J., Cobbold R., Hovde C.J. (2008). Low numbers of intestinal Shiga toxin-producing *E. coli* correlate with a poor prognosis in sheep infected with bovine leukemia virus. J. Vet. Sci..

[207] Steinberg K.M., Levin B.R. (2007). Grazing protozoa and the evolution of the *Escherichia coli* O157:H7 Shiga toxin-encoding prophage. Proc. Biol. Sci..

[208] Schmidt C.E., Shringi S., Besser T.E. (2016). Protozoan Predation of *Escherichia coli* O157:H7 Is Unaffected by the Carriage of Shiga Toxin-Encoding Bacteriophages. PLoS ONE.

[209] George A.S., Rehfuss M.Y.M., Parker C.T., Brandl M.T. (2020). The transcriptome of *Escherichia coli* O157: H7 reveals a role for oxidative stress resistance in its survival from predation by Tetrahymena. FEMS Microbiol. Ecol..

[210] Chekabab S.M., Daigle F., Charette S.J., Dozois C.M., Harel J. (2013). Shiga toxins decrease enterohaemorrhagic *Escherichia coli* survival within Acanthamoeba castellanii. FEMS Microbiol. Lett..

[211] Matthews L., McKendrick I.J., Ternent H., Gunn G.J., Synge B., Woolhouse M.E. (2006). Super-shedding cattle and the transmission dynamics of *Escherichia coli* O157. Epidemiol. Infect..

[212] Robinson S.E., Brown P.E., Wright E.J., Hart C.A., French N.P. (2009). Quantifying within- and between-animal variation and uncertainty associated with counts of *Escherichia coli* O157 occurring in naturally infected cattle faeces. J. R. Soc. Interface.

[213] Cull C.A., Paddock Z.D., Nagaraja T.G., Bello N.M., Babcock A.H., Renter D.G. (2012). Efficacy of a vaccine and a direct-fed microbial against fecal shedding of *Escherichia coli* O157:H7 in a randomized pen-level field trial of commercial feedlot cattle. Vaccine.

[214] Murphy B.P., McCabe E., Murphy M., Buckley J.F., Crowley D., Fanning S., Duffy G. (2016). Longitudinal Study of Two Irish Dairy Herds: Low Numbers of Shiga Toxin-Producing *Escherichia coli* O157 and O26 Super-Shedders Identified. Front. Microbiol..

[215] Matthews L., Reeve R., Gally D.L., Low J.C., Woolhouse M.E., McAteer S.P., Locking M.E., Chase-Topping M.E., Haydon D.T., Allison L.J. (2013). Predicting the public health benefit of vaccinating cattle against *Escherichia coli* O157. Proc. Natl. Acad. Sci. USA.

[216] Cobbold R., Desmarchelier P. (2002). Horizontal transmission of Shiga toxin-producing *Escherichia coli* within groups of dairy calves. Appl. Environ. Microbiol..

[217] van Diemen P.M., Dziva F., Abu-Median A., Wallis T.S., van den Bosch H., Dougan G., Chanter N., Frankel G., Stevens M.P. (2007). Subunit vaccines based on intimin and Efa-1 polypeptides induce humoral immunity in cattle but do not protect against intestinal colonisation by enterohaemorrhagic *Escherichia coli* O157:H7 or O26:H. Vet. Immunol. Immunopathol..

[218] Dziva F., Vlisidou I., Crepin V.F., Wallis T.S., Frankel G., Stevens M.P. (2007). Vaccination of calves with EspA, a key colonisation factor of *Escherichia coli* O157:H7, induces antigen-specific humoral responses but does not confer protection against intestinal colonisation. Vet. Microbiol..

[219] McNeilly T.N., Naylor S.W., Mahajan A., Mitchell M.C., McAteer S., Deane D., Smith D.G., Low J.C., Gally D.L., Huntley J.F. (2008). *Escherichia coli* O157:H7 colonization in cattle following systemic and mucosal immunization with purified H7 flagellin. Infect. Immun..

[220] Kieckens E., Rybarczyk J., Barth S.A., Menge C., Cox E., Vanrompay D. (2017). Effect of lactoferrin on release and bioactivity of Shiga toxins from different *Escherichia coli* O157:H7 strains. Vet. Microbiol..

[221] Rybarczyk J., Khalenkow D., Kieckens E., Skirtach A.G., Cox E., Vanrompay D. (2019). Lactoferrin translocates to the nucleus of bovine rectal epithelial cells in the presence of *Escherichia coli* O157:H7. Vet. Res..

[222] Kieckens E., Rybarczyk J., De Zutter L., Duchateau L., Vanrompay D., Cox E. (2015). Clearance of *Escherichia coli* O157:H7 infection in calves by rectal administration of bovine lactoferrin. Appl. Environ. Microbiol..

[223] Kuribayashi T., Seita T., Fukuyama M., Furuhata K., Honda M., Matsumoto M., Seguchi H., Yamamoto S. (2006). Neutralizing activity of bovine colostral antibody against verotoxin derived from enterohemorrhagic *Escherichia coli* O157:H7 in mice. J. Infect. Chemother..

[224] Martorelli L., Garimano N., Fiorentino G.A., Vilte D.A., Garbaccio S.G., Barth S.A., Menge C., Ibarra C., Palermo M.S., Cataldi A. (2018). Efficacy of a recombinant Intimin, EspB and Shiga toxin 2B vaccine in calves experimentally challenged with *Escherichia coli* O157:H7. Vaccine.

[225] MacLeod D.L., Gyles C.L. (1991). Immunization of pigs with a purified Shiga-like toxin II variant toxoid. Vet. Microbiol..

[226] Hovde C.J., Calderwood S.B., Mekalanos J.J., Collier R.J. (1988). Evidence that glutamic acid 167 is an active-site residue of Shiga-like toxin I. Proc. Natl. Acad. Sci. USA.

[227] Yamasaki S., Furutani M., Ito K., Igarashi K., Nishibuchi M., Takeda Y. (1991). Importance of arginine at position 170 of the A subunit of Vero toxin 1 produced by enterohemorrhagic *Escherichia coli* for toxin activity. Microb. Pathog..

[228] Makino S., Watarai M., Tabuchi H., Shirahata T., Furuoka H., Kobayashi Y., Takeda Y. (2001). Genetically modified Shiga toxin 2e (Stx2e) producing *Escherichia coli* is a vaccine candidate for porcine edema disease. Microb. Pathog..

[229] Ohmura-Hoshino M., Yamamoto M., Yuki Y., Takeda Y., Kiyono H. (2004). Non-toxic Stx derivatives from *Escherichia coli* possess adjuvant activity for mucosal immunity. Vaccine.

[230] Ishikawa S., Kawahara K., Kagami Y., Isshiki Y., Kaneko A., Matsui H., Okada N., Danbara H. (2003). Protection against Shiga toxin 1 challenge by immunization of mice with purified mutant Shiga toxin 1. Infect. Immun..

[231] Santiago-Mateo K., Zhao M., Lin J., Zhang W., Francis D.H. (2012). Avirulent K88 (F4)+ *Escherichia coli* strains constructed to express modified enterotoxins protect young piglets from challenge with a virulent enterotoxigenic *Escherichia coli* strain that expresses the same adhesion and enterotoxins. Vet. Microbiol..

[232] Mayer C.L., Leibowitz C.S., Kurosawa S., Stearns-Kurosawa D.J. (2012). Shiga toxins and the pathophysiology of hemolytic uremic syndrome in humans and animals. Toxins.

[233] Cobbold R., Desmarchelier P. (2000). A longitudinal study of Shiga-toxigenic *Escherichia coli* (STEC) prevalence in three Australian diary herds. Vet. Microbiol..

[234] Martorelli L., Albanese A., Vilte D., Cantet R., Bentancor A., Zolezzi G., Chinen I., Ibarra C., Rivas M., Mercado E.C. (2017). Shiga toxin-producing *Escherichia coli* (STEC) O22:H8 isolated from cattle reduces E. coli O157:H7 adherence in vitro and in vivo. Vet. Microbiol..

[235] Carter M.Q. (2017). Decoding the Ecological Function of Accessory Genome. Trends Microbiol..

[236] Leopold S.R., Magrini V., Holt N.J., Shaikh N., Mardis E.R., Cagno J., Ogura Y., Iguchi A., Hayashi T., Mellmann A. (2009). A precise reconstruction of the emergence and constrained radiations of *Escherichia coli* O157 portrayed by backbone concatenomic analysis. Proc. Natl. Acad. Sci. USA.

[237] Caprioli A., Morabito S., Brugereb H., Oswald E. (2005). Enterohaemorrhagic *Escherichia coli*: Emerging issues on virulence and modes of transmission. Vet. Res..

[238] Naylor S.W., Gally D.L., Low J.C. (2005). Enterohaemorrhagic *E. coli* in veterinary medicine. Int. J. Med. Microbiol..

[239] Callaway T.R., Anderson R.C., Edrington T.S., Genovese K.J., Harvey R.B., Poole T.L., Nisbet D.J. (2004). Recent pre-harvest supplementation strategies to reduce carriage and shedding of zoonotic enteric bacterial pathogens in food animals. Anim. Health Res. Rev..

[240] Callaway T.R., Carr M.A., Edrington T.S., Anderson R.C., Nisbet D.J. (2009). Diet, *Escherichia coli* O157:H7, and cattle: A review after 10 years. Curr. Issues Mol. Biol..

[241] Farrokh C., Jordan K., Auvray F., Glass K., Oppegaard H., Raynaud S., Thevenot D., Condron R., De Reu K., Govaris A. (2013). Review of Shiga-toxin-producing *Escherichia coli* (STEC) and their significance in dairy production. Int. J. Food. Microbiol..

[242] Vande Walle K., Vanrompay D., Cox E. (2013). Bovine innate and adaptive immune responses against *Escherichia coli* O157:H7 and vaccination strategies to reduce faecal shedding in ruminants. Vet. Immunol. Immunopathol..

[243] Snedeker K.G., Campbell M., Sargeant J.M. (2012). A systematic review of vaccinations to reduce the shedding of *Escherichia coli* O157 in the faeces of domestic ruminants. Zoonoses Public Health.

[244] Van Donkersgoed J., Hancock D., Rogan D., Potter A.A. (2005). *Escherichia coli* O157:H7 vaccine field trial in 9 feedlots in Alberta and Saskatchewan. Can. Vet. J..

[245] Mahajan A., Currie C.G., Mackie S., Tree J., McAteer S., McKendrick I., McNeilly T.N., Roe A., La Ragione R.M., Woodward M.J. (2009). An investigation of the expression and adhesin function of H7 flagella in the interaction of *Escherichia coli* O157: H7 with bovine intestinal epithelium. Cell. Microbiol..

